# Supplementary figures and images for: Rivalry between pitch and timbre in auditory stream segregation
Source: PLoS One. 2025 Jun 5;20(6):e0323964. doi: 10.1371/journal.pone.0323964 (PMC12140245; doi:10.1371/journal.pone.0323964)

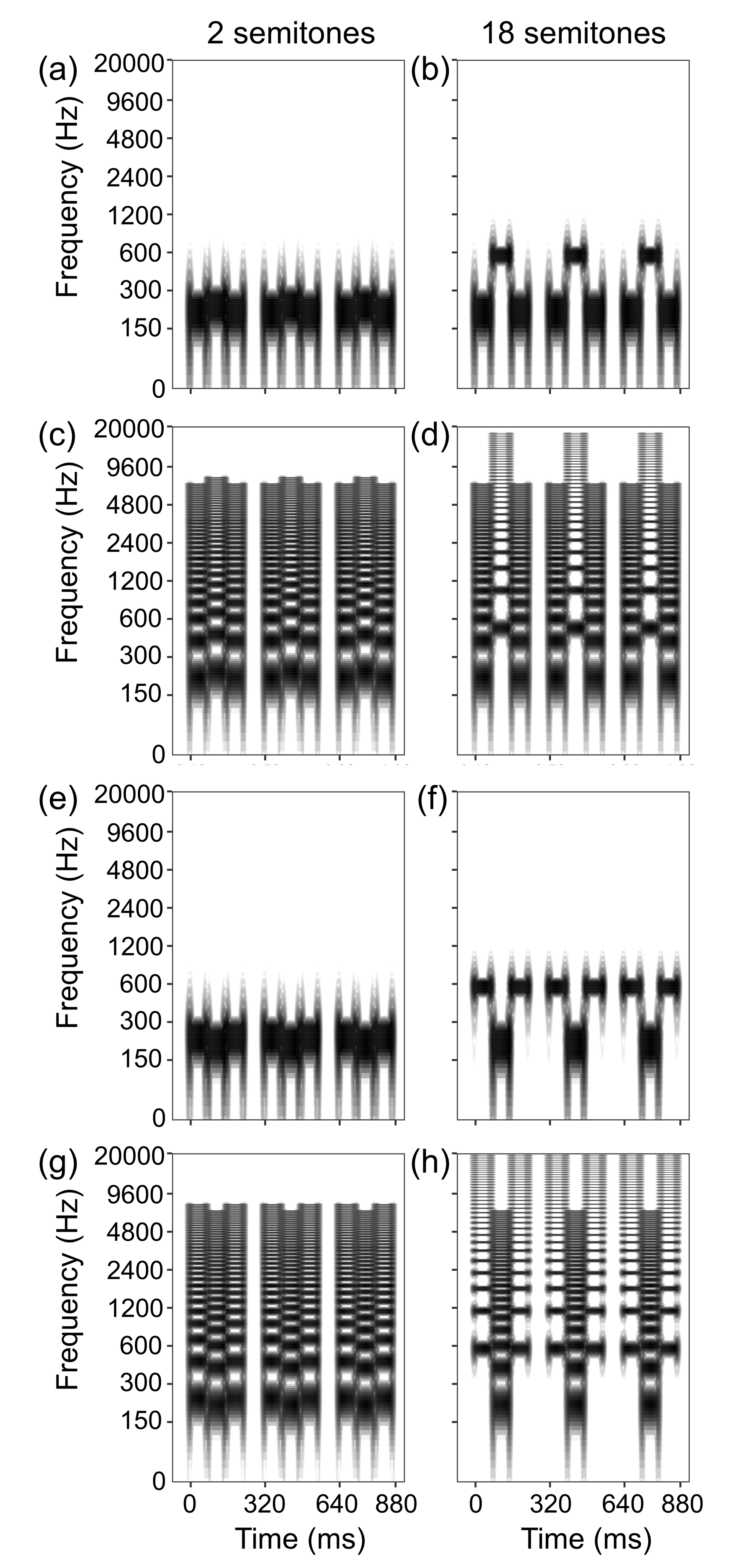

Supplement: S1 Fig — LHL_ triplets with pure tones and full-band harmonic complex tones (a–b and c–d) were used in both experiments 1 and 2. HLH_ triplets with pure tones and full-band harmonic complex tones (e–f and g–h) were used in both experiments 3 and 4. The left column represents exemplars with 2-semitone separation for explaining the one-stream concept, and the right column represents exemplars with 18-semitone separation for explaining the two-stream concept (TIF) [file pone.0323964.s001.tif]

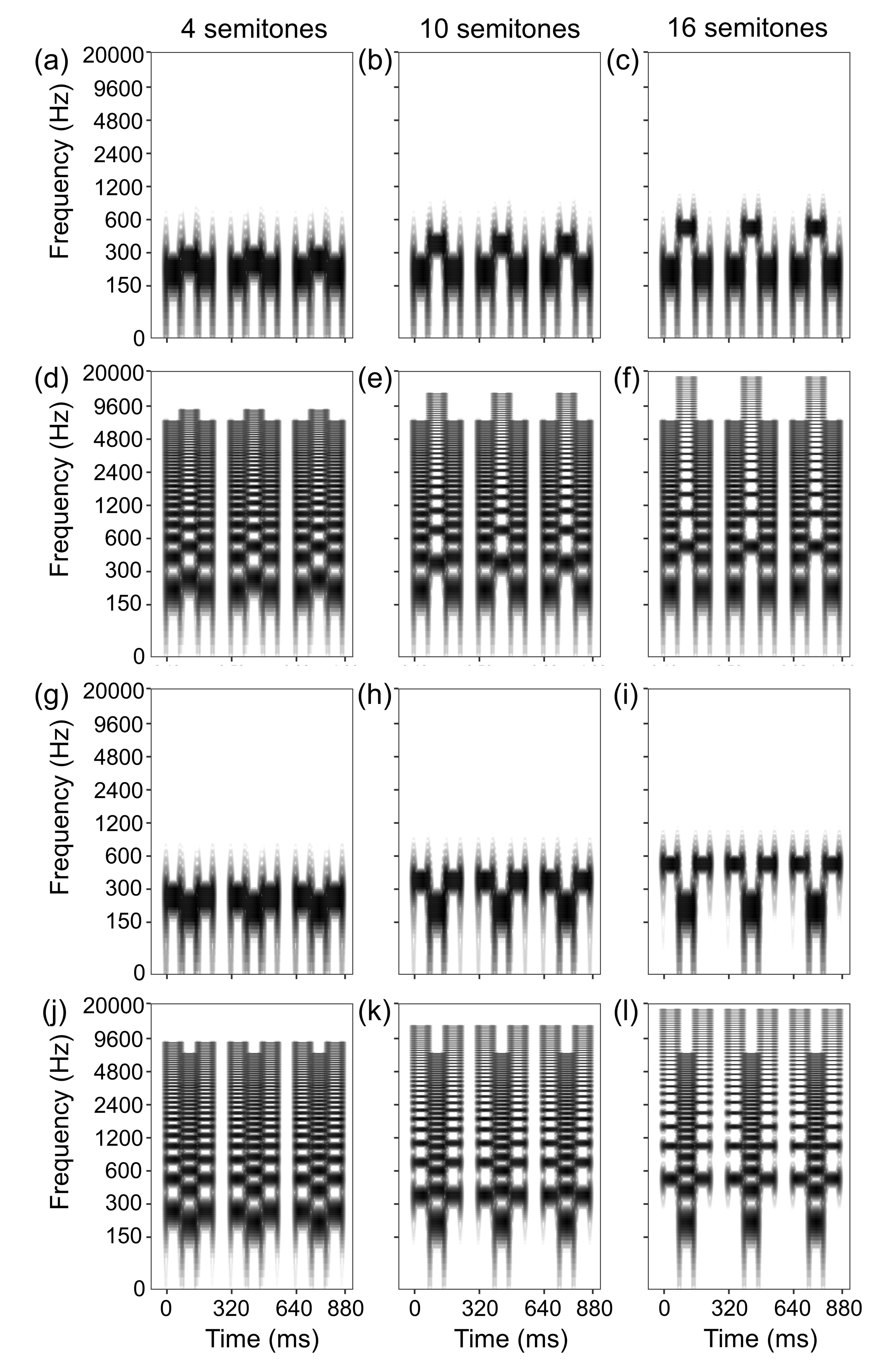

Supplement: S2 Fig — LHL_ triplets with pure tones and full-band harmonic complex tones (a–c and d–f) were used in both experiments 1 and 2. HLH_ triplets with pure tones and full-band harmonic complex tones (g–i and j–l) were used in both experiments 3 and 4. The columns are in the order of 4-, 10-, and 16-semitone separation from left to right. (TIF) [file pone.0323964.s002.tif]

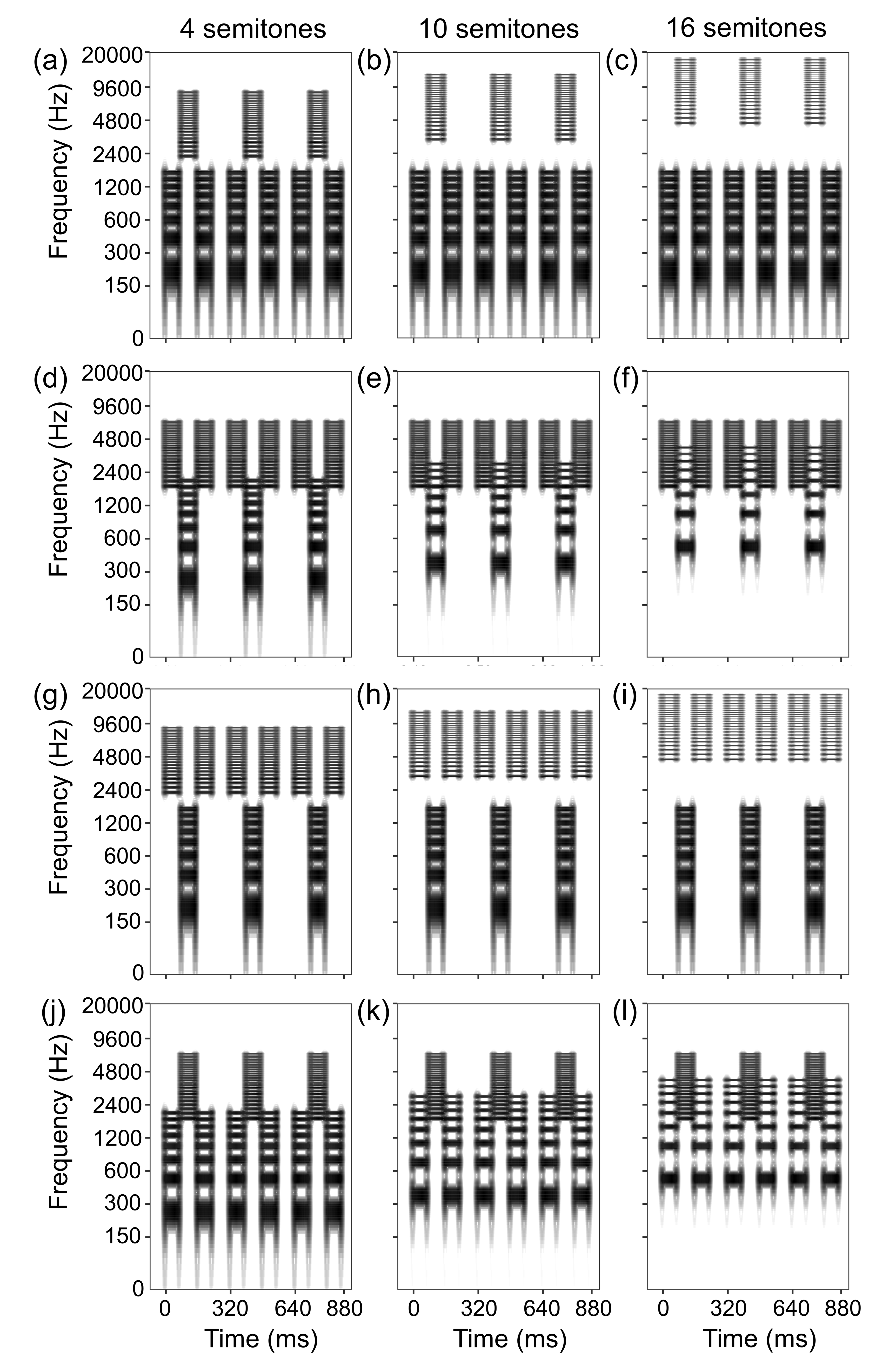

Supplement: S3 Fig — LHL_ triplets with congruent and incongruent stripe tones (a–c and d–f) were used in experiments 1 and 2. HLH_ triplets with congruent and incongruent stripe tones (g–i and j–l) were used in experiments 3 and 4. (TIF) [file pone.0323964.s003.tif]

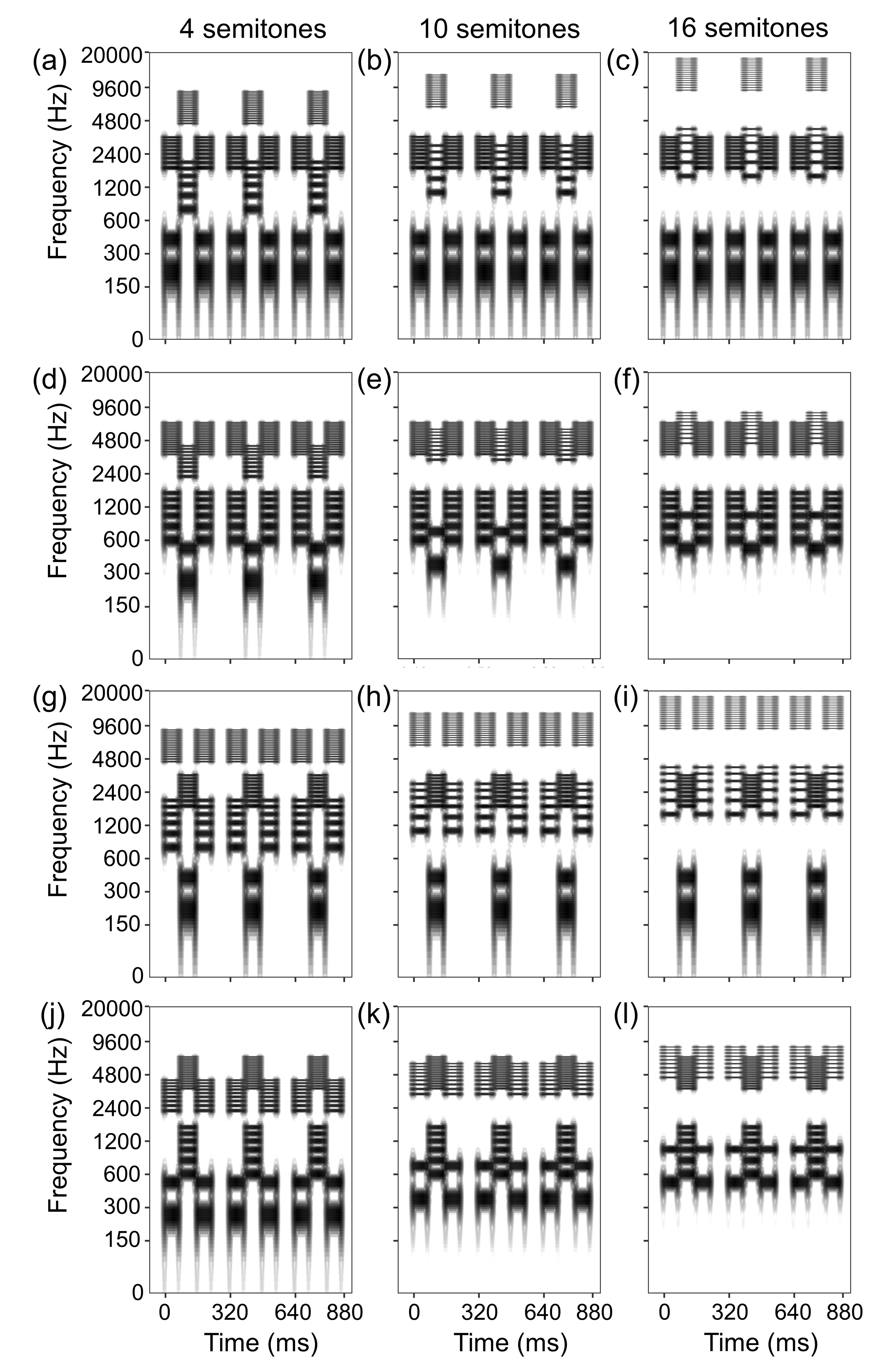

Supplement: S4 Fig — LHL_ triplets with congruent and incongruent stripe tones (a–c and d–f) were used in experiments 1 and 2. HLH_ triplets with congruent and incongruent stripe tones (g–i and j–l) were used in experiments 3 and 4. (TIF) [file pone.0323964.s004.tif]

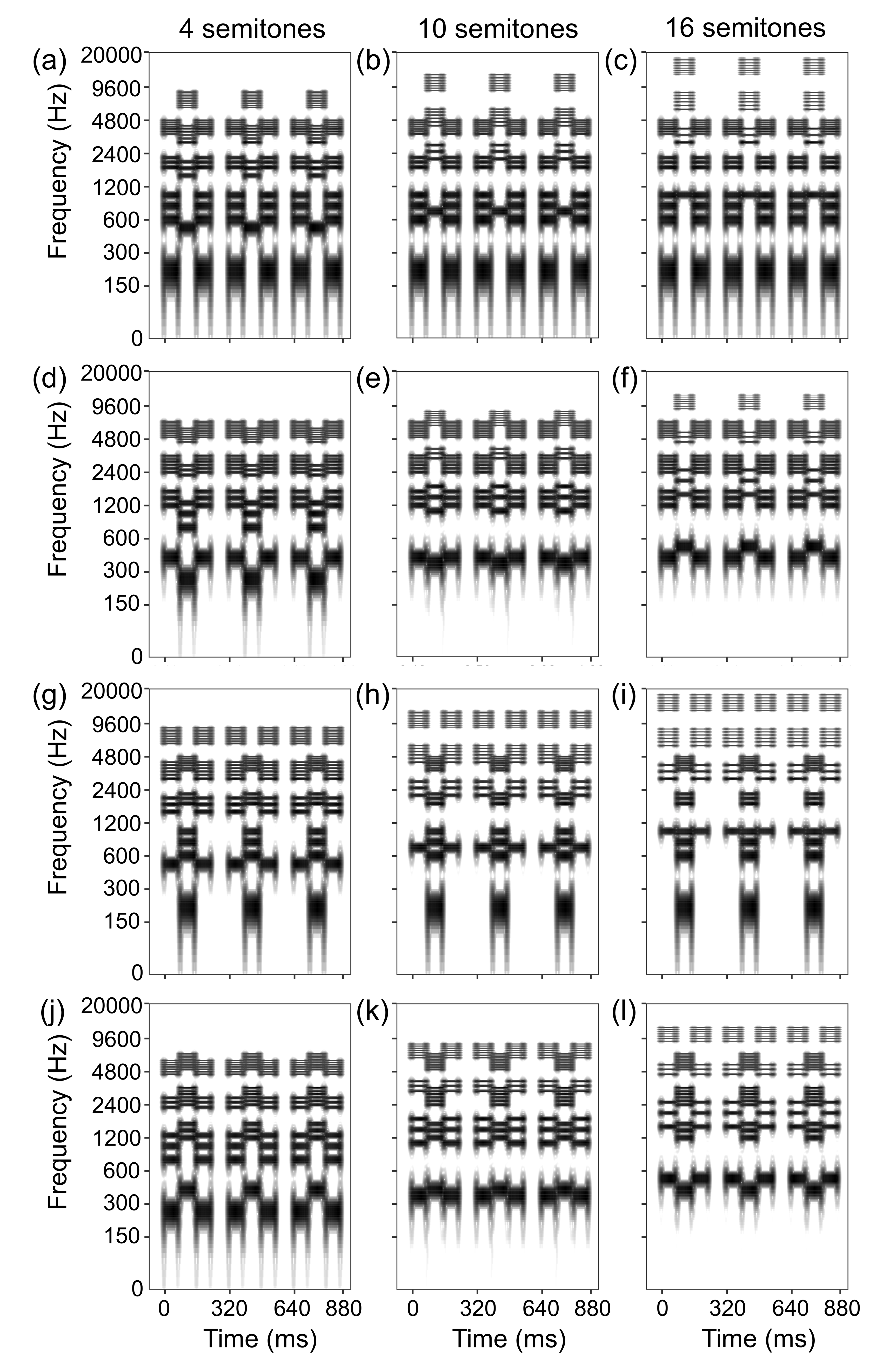

Supplement: S5 Fig — LHL_ triplets with congruent and incongruent stripe tones (a–c and d–f) were used in experiments 1 and 2. HLH_ triplets with congruent and incongruent stripe tones (g–i and j–l) were used in experiments 3 and 4. (TIF) [file pone.0323964.s005.tif]

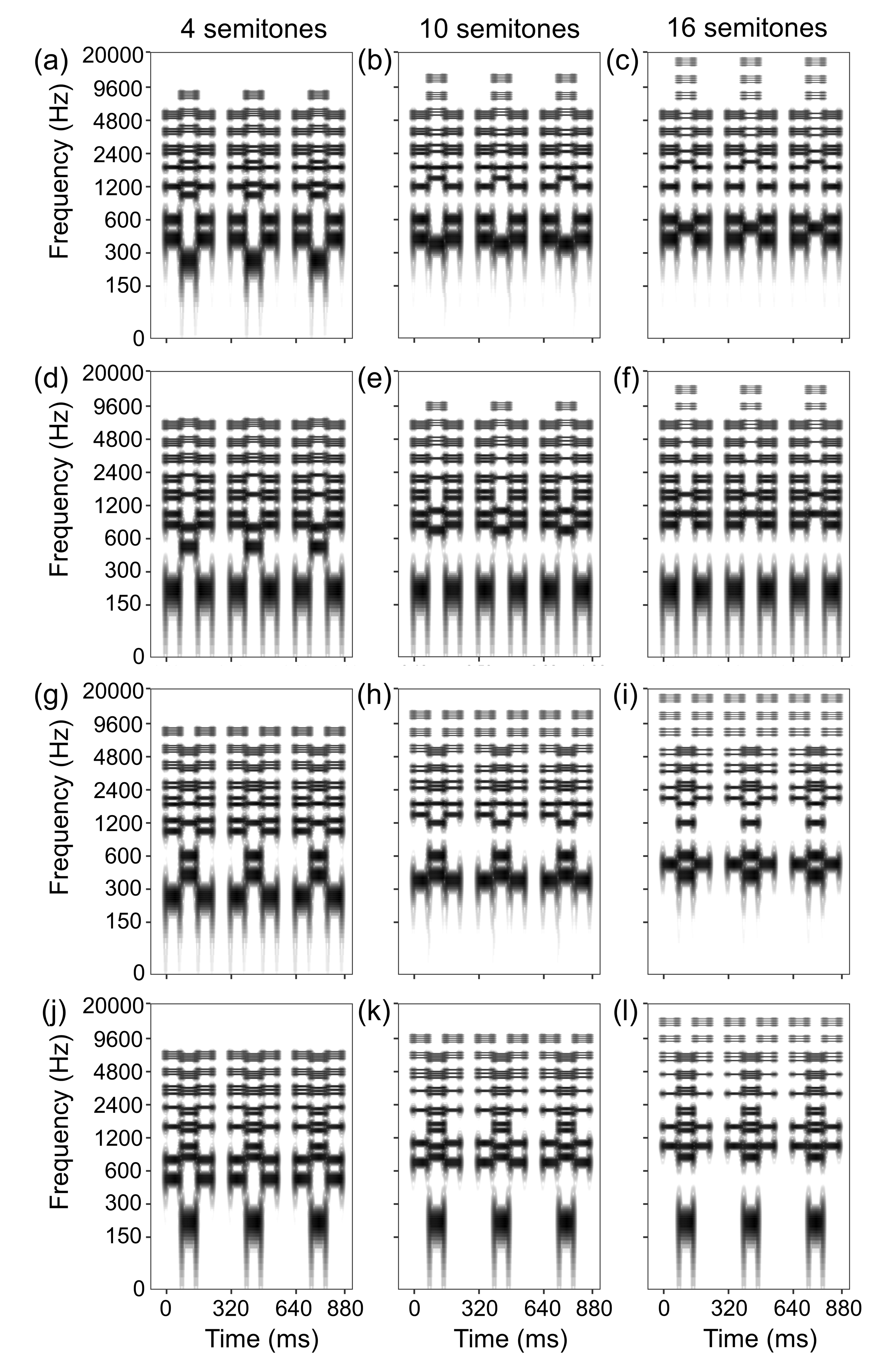

Supplement: S6 Fig — LHL_ triplets with congruent and incongruent stripe tones (a–c and d–f) were used in experiments 1 and 2. HLH_ triplets with congruent and incongruent stripe tones (g–i and j–l) were used in experiments 3 and 4. (TIF) [file pone.0323964.s006.tif]
