# Supplementary material for: Rivalry between pitch and timbre in auditory stream segregation
Source: PLoS One. 2025 Jun 5;20(6):e0323964. doi: 10.1371/journal.pone.0323964 (PMC12140245; doi:10.1371/journal.pone.0323964)
Supplement: S1 Table — (PDF) [file pone.0323964.s008.pdf]

S1 Table: GLMM analysis summary for the control conditions

Table S1a. Fit statistics and model summary

Fit statistics	
-2 Residual Log Pseudo-Likelihood	56803.2
Generalized χ^2	13569.35
Generalized χ^2/df	1.67
**Model summary**	
Response	2 streams (0 or 1)
Distribution	Binomial
Link function	Logit

Table S1b. Random effects covariance parameter estimates

Variance component	Estimate	Std error	95% lower	95% upper	Wald p
Trial block nested under participant	1.485	0.16	1.171	1.798	< 0.001
Congruency	0.506	0.72	-0.905	1.918	0.482

Table S1c. Fixed effect tests

Effect	df	F	p $(Prob > F)$	Statistical power
Control stimulus type (S)	$(1, 8148)$	44.92	< 0.001	0.886
(Fundamental) frequency separation (F)	$(2, 8148)$	689.644	< 0.001	0.998
Tone sequence pattern (T)	$(1, 8148)$	2.561	0.11	0.327
S×F	$(2, 8148)$	6.334	0.002	0.731
S×T	$(1, 8148)$	3.454	0.063	0.392
F×T	$(2, 8148)$	1.303	0.272	0.244
S×F×T	$(2, 8148)$	0.058	0.944	0.048

Table S1d. Fixed effects parameter estimates

Term	Estimate	Std error	df (denominator)	t ratio	p $(Prob > \|t\|)$	95% lower	95% upper
Intercept	-1.516	0.515	1.1	-2.941	0.192	-6.927	3.895
Control stimulus type (S)[pure tone]	-0.598	0.089	8148	-6.702	< 0.001	-0.773	-0.423
(Fundamental) frequency separation (F)[4]	-4.297	0.176	8148	-24.4	< 0.001	-4.642	-3.952
F[10]	0.951	0.094	8148	10.148	< 0.001	0.767	1.135
Tone sequence pattern (T)[HLH]	0.143	0.089	8148	1.6	0.11	-0.032	0.317
S[pure tone]×F[4]	0.614	0.173	8148	3.557	< 0.001	0.276	0.953
S[pure tone]×F[10]	-0.308	0.093	8148	-3.302	0.001	-0.491	-0.125
S[pure tone]×T[HLH]	0.165	0.089	8148	1.859	0.063	-0.009	0.34
F[4]×T[HLH]	-0.272	0.173	8148	-1.577	0.115	-0.611	0.066
F[10]×T[HLH]	0.147	0.093	8148	1.581	0.114	-0.035	0.33
S[pure tone]×F[4]×T[HLH]	-0.036	0.173	8148	-0.207	0.836	-0.374	0.303
S[pure tone]×F[10]×T[HLH]	0.028	0.093	8148	0.298	0.765	-0.155	0.21
