# Supplementary material for: Rivalry between pitch and timbre in auditory stream segregation
Source: PLoS One. 2025 Jun 5;20(6):e0323964. doi: 10.1371/journal.pone.0323964 (PMC12140245; doi:10.1371/journal.pone.0323964)
Supplement: S2 Table — (PDF) [file pone.0323964.s009.pdf]

S2 Table: GLMM analysis summary for the stripe-tone conditions

Table S2a. Fit statistics and model summary

Fit statistics	
-2 Residual Log Pseudo-Likelihood	132565.59
Generalized χ^2	10972.95
Generalized χ^2/df	0.67
Model summary	
Response	2 streams (0 or 1)
Distribution	Binomial
Link function	Logit

Table S2b. Random effects covariance parameter estimates

Variance component	Estimate	Std error	95% lower	95% upper	Wald p
Trial block nested under participant	1.622	0.166	1.296	1.948	< 0.001
Experiment order (1, 2, 4, and 3 or 2, 1, 3, and 4 in this order)	0.19	0.285	-0.368	0.748	0.505

Table S2c. Fixed effect tests

Effect	df	F	p $(Prob > F)$	Statistical power
Congruency (C)	$(1, 16272)$	0.012	0.912	< 0.001
Number of bands (N)	$(3, 16272)$	0.049	0.986	0.034
Fundamental frequency separation (F)	$(2, 16272)$	0.031	0.97	< 0.001
Tone sequence pattern (T)	$(1, 16272)$	0.001	0.976	< 0.001
C×N	$(3, 16272)$	0.019	0.996	< 0.001
C×F	$(2, 16272)$	0.004	0.996	< 0.001
C×T	$(1, 16272)$	0.017	0.897	< 0.001
N×F	$(6, 16272)$	9.601	< 0.001	0.874
N×T	$(3, 16272)$	0.002	> 0.999	< 0.001
F×T	$(2, 16272)$	0.002	0.998	< 0.001
C×N×F	$(6, 16272)$	22.524	< 0.001	0.926
C×N×T	$(3, 16272)$	0.007	0.999	< 0.001
C×F×T	$(2, 16272)$	0.004	0.996	< 0.001
N×F×T	$(6, 16272)$	0.005	> 0.999	< 0.001
C×N×F×T	$(6, 16272)$	0.007	> 0.999	< 0.001

Table S2d. Fixed effects parameter estimates

Term	Estimate	Std error	*df* (denominator)	*t* ratio	*p* (*Prob* > |*t*|)	95% lower	95% upper
Intercept	4.945	11.048	16272	0.448	0.655	-16.711	26.601
Congruency (C)[congruent]	1.22	11.044	16272	0.11	0.912	-20.427	22.868
Number of bands (N)[2]	5.434	28.617	16272	0.19	0.849	-50.658	61.526
N[4]	0.319	16.167	16272	0.02	0.984	-31.369	32.008
N[8]	-1.787	16.167	16272	-0.111	0.912	-33.475	29.901
Fundamental frequency separation (F)[4]	-2.73	11.044	16272	-0.247	0.805	-24.377	18.918
F[10]	0.622	16.697	16272	0.037	0.97	-32.106	33.349
Tone sequence pattern (T)[HLH]	0.334	11.044	16272	0.03	0.976	-21.313	21.982
C[congruent]×N[2]	-0.166	28.617	16272	-0.006	0.995	-56.258	55.926
C[congruent]×N[4]	0.388	16.167	16272	0.024	0.981	-31.301	32.077
C[congruent]×N[8]	1.544	16.167	16272	0.096	0.924	-30.144	33.233
C[congruent]×F[4]	-1.045	11.044	16272	-0.095	0.925	-22.693	20.602
C[congruent]×F[10]	0.266	16.697	16272	0.016	0.987	-32.461	32.993
C[congruent]×T[HLH]	1.433	11.044	16272	0.13	0.897	-20.214	23.08
N[2]×F[4]	-2.079	28.617	16272	-0.073	0.942	-58.171	54.014
N[2]×F[10]	3.108	47.225	16272	0.066	0.948	-89.458	95.675
N[4]×F[4]	1.463	16.167	16272	0.09	0.928	-30.226	33.152
N[4]×F[10]	-1.29	20.45	16272	-0.063	0.95	-41.374	38.794
N[8]×F[4]	0.503	16.167	16272	0.031	0.975	-31.186	32.191
N[8]×F[10]	-1.36	20.449	16272	-0.067	0.947	-41.443	38.723
N[2]×T[HLH]	-1.505	28.617	16272	-0.053	0.958	-57.597	54.586
N[4]×T[HLH]	0.642	16.167	16272	0.04	0.968	-31.047	32.33
N[8]×T[HLH]	0.788	16.167	16272	0.049	0.961	-30.9	32.476
F[4]×T[HLH]	-0.36	11.044	16272	-0.033	0.974	-22.007	21.288
F[10]×T[HLH]	-0.844	16.697	16272	-0.051	0.96	-33.572	31.883
C[congruent]×N[2]×F[4]	-0.053	28.617	16272	-0.002	0.999	-56.146	56.039
C[congruent]×N[2]×F[10]	1.609	47.225	16272	0.034	0.973	-90.957	94.175
C[congruent]×N[4]×F[4]	-0.943	16.167	16272	-0.058	0.954	-32.632	30.746
C[congruent]×N[4]×F[10]	-0.856	20.45	16272	-0.042	0.967	-40.939	39.228
C[congruent]×N[8]×F[4]	-0.873	16.167	16272	-0.054	0.957	-32.562	30.815
C[congruent]×N[8]×F[10]	-0.492	20.449	16272	-0.024	0.981	-40.575	39.591
C[congruent]×N[2]×T[HLH]	1.574	28.617	16272	0.055	0.956	-54.517	57.666
C[congruent]×N[4]×T[HLH]	-0.022	16.167	16272	-0.001	0.999	-31.711	31.666
C[congruent]×N[8]×T[HLH]	-0.078	16.167	16272	-0.005	0.996	-31.766	31.61
C[congruent]×F[4]×T[HLH]	-0.927	11.044	16272	-0.084	0.933	-22.575	20.72
C[congruent]×F[10]×T[HLH]	-0.385	16.697	16272	-0.023	0.982	-33.113	32.342
N[2]×F[4]×T[HLH]	1.225	28.617	16272	0.043	0.966	-54.868	57.318
N[2]×F[10]×T[HLH]	-0.914	47.225	16272	-0.019	0.985	-93.48	91.652
N[4]×F[4]×T[HLH]	-0.743	16.167	16272	-0.046	0.963	-32.432	30.946
N[4]×F[10]×T[HLH]	-0.027	20.45	16272	-0.001	0.999	-40.11	40.057
N[8]×F[4]×T[HLH]	-0.65	16.167	16272	-0.04	0.968	-32.338	31.039
N[8]×F[10]×T[HLH]	0.044	20.449	16272	0.002	0.998	-40.039	40.127
C[congruent]×N[2]×F[4]×T[HLH]	-1.569	28.617	16272	-0.055	0.956	-57.661	54.524
C[congruent]×N[2]×F[10]×T[HLH]	0.307	47.225	16272	0.007	0.995	-92.259	92.874
C[congruent]×N[4]×F[4]×T[HLH]	0.333	16.167	16272	0.021	0.984	-31.356	32.022
C[congruent]×N[4]×F[10]×T[HLH]	-0.314	20.45	16272	-0.015	0.988	-40.397	39.77
C[congruent]×N[8]×F[4]×T[HLH]	0.112	16.167	16272	0.007	0.995	-31.577	31.8
C[congruent]×N[8]×F[10]×T[HLH]	-0.518	20.449	16272	-0.025	0.98	-40.601	39.565
