# Supplementary material for: Rivalry between pitch and timbre in auditory stream segregation
Source: PLoS One. 2025 Jun 5;20(6):e0323964. doi: 10.1371/journal.pone.0323964 (PMC12140245; doi:10.1371/journal.pone.0323964)
Supplement: Attachment — Submitted filename: comments.pdf [file pone.0323964.s010.pdf]

Review comments

- Major issues:
  - Lack of clear research motivations, research questions, hypothesis and predictions.
  - Insufficient discussion
  - A number of redundant expressions and inconsistent expressions that confused the readers
- Introduction
  - Is 'F segregation' in pure tone sequences and 'F0 segregation' in complex tone sequences fundamentally different or the same?
  - The introduction of timbre as a perceptual cue for segregation is vague and lacks logical consistency (p.4, l.53). The concept of timbre is not introduced correctly, and the author even uses 'spectral difference' instead of 'timbral difference' in the next paragraph.
  - More discussion is needed on how previous studies have addressed the combined effect of F0 separation and spectral differences. More importantly, the authors need to clearly state their motivation for investigating this combined effect.
  - There is a lack of rationale for conducting research on non-speech stimuli. Based on the review, research on speech stimuli has not yet reached a solid conclusion. Why didn't the authors continue to work on speech stimuli?
  - There is no clear introduction to the concept of 'switching frequency bands' or the rationale for choosing it as a representative of timbral cues. (p.5. l.83).
  - Again, what is 'effect of congruency' and the motivation of including it as a variable.
  - The research lacks clear questions, hypotheses, and predictions. Given the number of variables and interactions explored, the study seems more exploratory rather than hypothesis-driven.
- Methods
  - The authors specifically mentioned that some participants attended Technical Listening Training classes (p.5, l.102-108), but they did not discuss how this training might have influenced the results.
  - The language background of the participants is missing.
  - Too much information in 'Conditions'—please add figures to facilitate understanding.
  - The variables from control conditions and experimental conditions

seem incomparable. The ‘stimuli type’ in control condition and ‘number of frequency bands’ in experimental condition appear to refer to the same thing—the authors should consider making their names consistent. This issue occurs throughout the manuscript.

- The procedure section is lengthy, disorganized, and not reader-friendly. Some information overlaps with details about the stimuli (e.g., p.10, l.183-186). Please explain the procedure briefly and clearly. Consider adding a flowchart and a summary.
- P10 l.191-193: Does this mean that they learned the concept during instruction using stimuli that were not used in the experiment, and then had to apply that concept on their own during the experiment? If so, it seems similar to standard instructions used in many perception experiments. Since the authors mentioned it specifically and in great detail, I’m wondering if there is a particular reason for that.
- P.11 l. 215-216: For participants who cancelled and re-entered their responses, which response did the authors use for the final analysis—the first one or the re-entered one? Were they allowed to hear the stimuli a second time? Why did they set up a confirmation box here?

- Results
  - For experimental condition, since the model revealed significant two-way and three-way interaction, I am wondering if the authors conducted any post-hoc analysis?
- Discussions
  - I am not clear about the reasoning for keeping section 4.1 in the discussion —it sounds more like a justification of the methods , which would be more appropriate in the methods section.
  - The title of 4.2 is simply a collection of words rather than a summary of content.
  - Half of section 4.2 repeats the results rather than discussing them.
  - I don’t see the purpose of introducing the spectral centroid starting from p18, l. 399. It seems like the authors are introducing new concepts and results in the discussion, making the study feel somewhat ad hoc and overly exploratory.
  - I noticed that musical training background wasn't controlled for or considered as a predictive factor in this study, yet the authors suddenly introduce it (starting from l. 406) and spend quite a few lines expanding on this point.
- Conclusions

- Please state the theoretical significance and potential social contribution of the study.
