# Supplementary material for: Rivalry between pitch and timbre in auditory stream segregation
Source: PLoS One. 2025 Jun 5;20(6):e0323964. doi: 10.1371/journal.pone.0323964 (PMC12140245; doi:10.1371/journal.pone.0323964)
Supplement: Attachment — Submitted filename: Response Letter.pdf [file pone.0323964.s011.pdf]

Point-by-point responses to the Reviewers' comments

Geng-Yan Jhang, Kazuo Ueda, Hiroshige Takeichi, Gerard B. Remijn, and Emi Hasuo

28 February 2025

Ref.: PONE-D-24-04194, "Rivalry between pitch and timbre in auditory stream segregation," previously entitled "Rivalry between fundamental frequency separation and switching frequency bands for auditory stream segregation," by Geng-Yan Jhang, Kazuo Ueda, Hiroshige Takeichi, Gerard B. Remijn, and Emi Hasuo.

Thank you very much for the constructive comments on the above manuscript. We have revised it according to the suggestions. Line numbers in the previous version of the manuscript are indicated in this response letter. Additionally, *current line numbers*, referring to the line numbers in the marked-up revised manuscript, are indicated where it is necessary. We hope the revised manuscript meets the Editor's and Reviewers' expectations.

1 Reply to Reviewer 1

1.1 General Comments

> This paper addresses an important topic in the area of acoustics. I really appreciate the careful consideration the authors have given to the experimental design, which is clearly described and executed. To make the paper compelling, there are some areas where further clarification or additional detail could be beneficial, which I have uploaded as an attachment.

Response We are pleased to note your favorable comment in the opening sentence. Thank you for providing helpful suggestions. We have made revisions as you suggested.

1.2 Major issues

1. Lack of clear research motivations, research questions, hypothesis, and predictions.
2. Insufficient discussion.
3. A number of redundant expressions and inconsistent expressions that confused the readers.

Response Thank you for the summary of the issues. We have revised the manuscript based on your specific comments.

1.3 Comments on the Introduction

1. Is 'F segregation' in pure tone sequences and 'F0 segregation' in complex tone sequences fundamentally different or the same?

Response We used "F separation" and "F0 separation" to represent a "(fundamental) frequency difference between a low-pitch tone and a high-pitch tone, expressed in semitone units." Still, we never used "F *segregation*" and "F0 *segregation*." To avoid any potential confusion, the following revisions were made.

(a) "(F)" and "(F_0)" were removed.

(b) "F" was replaced with "frequency".

(c) "F_0" was replaced with "fundamental frequency".

(d) "F or F_0" was replaced with "(fundamental) frequency".

(e) The following definition of "(fundamental) frequency separation" was added in current lines 40–43.

> In this connection, we define a "(fundamental) frequency separation" as a "(fundamental) frequency difference between a low-pitch tone and a high-pitch tone, expressed in semitone units" in this article.

2. The introduction of timbre as a perceptual cue for segregation is vague and lacks logical consistency (p.4, l.53). The concept of timbre is not introduced correctly, and the author even uses 'spectral difference' instead of 'timbral difference' in the next paragraph.

Response Thank you for your comments. We realized that the Introduction, title, and abstract must be revised to address your comments and properly introduce pitch and timbre. We made the following revisions.

Title: We revised the previous title, "Rivalry between fundamental frequency separation and switching frequency bands for auditory stream segregation" as "Rivalry between pitch and timbre in auditory stream segregation".

Abstract: (a) The first three sentences were revised as follows.

Previous version

It has been shown that a tone sequence of alternating two tones may elicit perceptually distinct two streams (*sequential stream segregation*), depending on fundamental frequency (F_0) separations (closely related to pitch differences) and spectral envelope differences (closely related to timbre differences). We further explored how F_0 separation (a pitch cue) interacted with switching frequency bands (a timbral cue) in sequential stream segregation to elucidate auditory organization (integration and segregation) for tone sequences with frequency components dispersed over the frequency and time domains. For each sequence, harmonic complex tones were divided into 2, 4, 8, or 16 frequency bands, frequency components (i.e., harmonics) in every other band were removed, and the resulting two possible stripe-like patterns of stimulus spectrograms were alternated.

Revised version

Two rapidly alternating tones with different pitches may be perceived as one integrated stream when the pitch differences are small or two separated streams when the pitch differences are large. Likewise, timbre differences between two tones may also cause such *sequential stream segregation*. Moreover, the effects of pitch and timbre on stream segregation can cancel each other out, which is called a trade-off. However, how timbre differences caused by specific patterns of spectral

shapes interact with pitch differences and affect stream segregation has been largely unexplored. Therefore, we used *stripe tones*, in which stripe-like spectral patterns of harmonic complex tones were realized by grouping harmonic components into several bands based on harmonic numbers and removing harmonic components in every other band.

(b) ", irrespective of the combinations of F_0 separations (4–16 semitones) and stripe-like patterns" was replaced with "against pitch proximity".

(c) "By contrast, F_0" was replaced with "By contrast, pitch"

(d) "came in between these two extremes, showing the most prominent effects of rivalry between F_0 separations and stripe-like patterns" was replaced with "most clearly showed the trade-off between pitch and timbre on stream segregation".

(e) "small number ($\leq$4) of frequency bands" was replaced with "small number ($\leq$4) of bands".

(f) "in blocks of harmonics. Furthermore, the results also suggest" was replaced with "between stripe-like spectral patterns, and"

(g) "has limits" was replaced with "looks to be limited"

(h) "harmonics distinctly" was replaced with "frequency components".

(i) "with a small number of frequency bands" was removed.

Lines 2–19: The beginning of the Introduction was replaced with the following (current lines 19–43).

Previous version

In our daily lives, we have to group certain frequency (F) components into several objects from a mixture of sounds (integration) and reject others (segregation). Moreover, we have to group a series of sounds into a coherent perceptual stream, which is supposed to come from the same sound source. These general processes of auditory perceptual organization have been referred to as *auditory scene analysis* [1], including auditory grouping for sequential sounds (e.g., Moore and Gockel [2, 3], Oh et al. [4], Rajasingam et al. [5], Roberts and Haywood [6], and Thomassen et al. [7]) and concurrent sounds (e.g., Gohari et al. [8] and Micheyl and Oxenham [9]). In particular, organizing sequential sounds into two (or more) groups of separate streams has been referred to as *auditory stream segregation* and has been investigated in laboratory experiments with sequentially presented sounds (*sequential stream segregation*).

The majority of the previous investigations on sequential stream segregation have employed pure tones (e.g., Anstis and Saida [10], Bregman and Campbell [11], Bregman [12], Haywood and Roberts [13–15], Miller and Heiser [16], Rajasingam et al. [5, 17], Rankin et al. [18], and van Noorden [19]). Specifically, tone triplet sequences with low-high-low (LHL-) and high-low-high (HLH-) tone triplet patterns have been widely used (Fig 1). Here, L has a frequency (F) lower than that of H, and "-" signifies a silent gap.

Revised version

Auditory scene analysis [1] refers to the auditory function in which we perceptually organize the outer world filled with a mixture of sounds. Sometimes, we can exploit binaural cues to separate several sounds, but the cues

are not always available or practical. Even in such situations, normal-hearing listeners usually successfully pick up a target from the background sounds, like a talker's voice talking on a noisy railway platform over a phone. Linguistic cues should contribute a lot to segregating speech and background noise in such a situation; nevertheless, the perceptual characteristics of the auditory system should be involved in the segregation process. Furthermore, if both a target and background are nonspeech, like a warning signal in background noise, we must make use of other cues, such as pitch and timbre. The auditory system can organize and segregate a sound mixture into more than two streams with the pitch and timbre differences of the sounds. The current investigation focuses on how pitch and timbre interact in *auditory stream segregation* [2].

Miller and Heise [3] reported that two rapidly alternating pure tones (i.e., tones having only one frequency component and a sinusoidal waveform) with some slight frequency differences are heard as a trill, a continuously changing pitch pattern. However, the two tones with a wide frequency separation are perceived as two separate and unrelated melodies. Since then, *sequential stream segregation* has been a widely studied research topic [4–22]. In particular, low-high-low (LHL_) and high-low-high (HLH_) tone patterns, i.e., so-called *triplets* (Fig 1), are the standard research tools to investigate sequential stream segregation caused by pitch differences. Here, "L" signifies a "low-pitch tone," "H" a "high-pitch tone," and "_" a "silent gap". In this connection, we define a "(fundamental) frequency separation" as a "(fundamental) frequency difference between a low-pitch tone and a high-pitch tone, expressed in semitone units" in this article.

Lines 16–23: The descriptions of one- and two-stream percepts for an LHL_ sequences were improved (current lines 37–43 and 48–52). Also, to avoid confusion, "-" (a hyphen) denoting a gap was replaced with "_" (an underscore) throughout the manuscript.

Previous version

Specifically, tone triplet sequences with low-high-low (LHL-) and high-low-high (HLH-) tone triplet patterns have been widely used (Fig 1). Here, L has a frequency (F) lower than that of H, and "-" signifies a silent gap. For an example with the LHL- tone triplet sequence, when Ls are perceptually grouped with a short gap in between (L-L-L-L-L-L...) and Hs with a long gap in between (-H—H—H–...), these two streams are segregated. Whereas, if Ls and Hs are perceptually grouped into one stream, a galloping rhythm pattern (LHL-LHL-LHL-...) is perceived.

Revised version

In particular, low-high-low (LHL_) and high-low-high (HLH_) tone patterns, i.e., so-called *triplets* (Fig 1), are the standard research tools to investigate sequential stream segregation caused by pitch differences. Here, "L" signifies a "low-pitch tone," "H" a "high-pitch tone," and "_" a "silent gap". In this connection, we define a "(fundamental) frequency separation" as a "(fundamental) frequency difference between a low-pitch tone and a high-pitch tone, expressed in semitone units" in this article.

Suppose a series of "LHL_" triplets is presented. If the tone sequence is perceptually grouped, then a listener will hear one stream with a galloping

rhythm (LHL_LHL_LHL_). In contrast, if the tone sequence is perceptually segregated into two streams, a listener will hear a low-pitch-tone sequence with a short gap in between (L_L_L_...) and a high-pitch-tone sequence with a long gap in between (H___H___H___...).

Fig 1, caption: "low (L) and high (H) tones" was replaced with "low-pitch and high-pitch tones (Ls and Hs)".

Fig 1, caption: Double quotation marks were added for "silent gap".

Line 23: A new paragraph started from "One way to promote segregation" (current line 53).

Line 23: "One way to promote segregation" was replaced with "One possibility to increasing probability of segregation".

Line 24: "way" was replaced "possibility".

Line 25: "(*building-up* [10, 12])" was replaced with "to *build up* segregation [5, 6]".

Line 26: "showed the" was replaced with "reported a".

Line 27: "by examining" was replaced with "in".

Line 28: "(i.e., the last three tones)" was removed.

Lines 28–30: Revised as follows (current lines 59–61).

Previous version

> only three sets of triplets (their control conditions) may be segregated with prominent F separation [13–15, 18]. Haywood and Roberts [13–15] revealed

Revised version

> with only three sets of triplets, they found

Line 31: "around 75–90% segregation" was replaced with "segregation around 75%–90% of the time".

Line 33: "25–55%" was replaced with "25%–55%".

Lines 33–34: "Thus, the effect of F separation on sequential stream segregation was shown with this convenient paradigm of three sets of triplets." was removed.

Line 35: Revised as follows (current lines 67–68).

Previous version

> Some studies on sequential stream segregation have used harmonic complex tones.

Revised version

> Harmonic complex tones have been used in some studies on sequential stream segregation.

Lines 36–37: The second sentence of the third paragraph in the Introduction was revised to introduce harmonic complex tones, pitch of missing fundamental, and timbre as follows (current lines 70–77).

Previous version

> Both fundamental frequency (F_0) separation and spectral differences, which roughly correspond to pitch and timbre differences, affect stream segregation.

Revised version

A harmonic complex tone consists of a series of pure tones called harmonics with frequencies that are integer multiples of a fundamental frequency, i.e., the lowest frequency. The pitch of a harmonic complex tone usually corresponds to the pitch of the fundamental; however, even if the fundamental frequency component is missing, the pitch of the harmonic complex tone does not change. This is called the missing fundamental phenomenon. When one modifies the level balance of frequency components in a harmonic complex tone, timbre varies, although this is not the only factor that affects the timbre of a harmonic complex tone.

Lines 37–38: A new paragraph started from "F_0 separation is a prominent cue" (current line 78).

Line 38: The reference "Shearer *et al.* (2018)" was removed.

Lines 38–42: The detailed descriptions of resolved harmonics were removed (current lines 79–80).

Previous version

clear pitches [20–22]: Pitch salience was enhanced with the resolved harmonic components [20] (the first 5–8 harmonics resolved on the basilar membrane [23, 24] within the inner ear, where corresponding clear peaks of excitation patterns were evoked [24]) or the low-numbered harmonics per se [21].

Revised version

clear pitches with resolvable harmonics, that is, usually the first five to eight harmonics [23, 24].

Lines 42–43: "F_0 separation promotes segregation, especially when the F_0 variations are correlated with spectral differences [25]." was removed.

Lines 43–45: The descriptions of stream segregation with unresolved harmonics were revised as follows (current lines 85–88).

Previous version

even without prominent spectral differences, F_0 separation between harmonic complex tones consisting of only unresolved harmonics induces stream segregation [20, 26–28].

Revised version

even without resolvable harmonics, fundamental frequency separation between bandpass-filtered harmonic complex tones induces stream segregation [23, 25–27].

Lines 46–47: Pitch-based segregation with amplitude-modulated noises was introduced in a clearer manner (current lines 89–91).

Previous version

Listeners perceive pitches in amplitude-modulated noises, thus they can perceive stream segregation with such stimuli, too [29–31].

Revised version

A sequence of amplitude-modulated noises may be perceptually segregated based on pitch separation [28–30].

Line 47: "On the other hand," was replaced with "Other factors related to timbre can influence stream segregation:", starting a new paragraph (current line 92).

Line 51: ", can affect stream segregation" was removed.

Lines 51–55: "In a similar vein, narrowband noises with different center frequencies can result in stream segregation as well [31, 39–42]. Thus, timbre is supposed to be another perceptual cue to induce segregation. Other findings of timbre-based segregation with real musical instrument sounds [43, 44] and synthesized musical instrument sounds [45–47] also support the claim." was removed.

3. More discussion is needed on how previous studies have addressed the combined effect of F0 separation and spectral differences. More importantly, the authors need to clearly state their motivation for investigating this combined effect.

4. There is a lack of rationale for conducting research on non-speech stimuli. Based on the review, research on speech stimuli has not yet reached a solid conclusion. Why didn't the authors continue to work on speech stimuli?

5. There is no clear introduction to the concept of 'switching frequency bands' or the rationale for choosing it as a representative of timbral cues. (p.5. l.83).

6. Again, what is 'effect of congruency' and the motivation of including it as a variable.

7. The research lacks clear questions, hypotheses, and predictions. Given the number of variables and interactions explored, the study seems more exploratory rather than hypothesis-driven.

Response These five points are mutually related. In essence, you require clarifying the rationale and research questions. The following revisions were made.

Lines 56–58: Revised as follows (current lines 102–104).

Previous version

A few studies have focused on the combined effects of F_0 separation (pitch differences) and spectral differences (timbral differences) on sequential stream segregation.

Revised version

Some studies have focused on the combined effects of pitch and timbre differences on sequential stream segregation.

Line 59: We added the following to summarize previous findings and define the term *trade-off* (current lines 105–107).

Thus, the effects of pitch and timbre on stream segregation can cancel each other out. We call this relationship hereafter a *trade-off* between pitch and timbre in stream segregation.

Line 59: "previous" was removed.

Lines 59–60: "(by spectral envelope)" was removed.

Lines 60–62: Revised as follows (current lines 109–111).

Previous version

controlled in relatively simple manners: a set of four consecutive harmonics was shifted [25], a spectral peak was moved [35], or the overall spectral slope was tilted [4].

Revised version

manipulated by shifting a set of four consecutive harmonics [7], moving a spectral peak [32], and tilting the overall spectral slope [34].

Lines 62–78: The following paragraphs replace the part as new paragraphs, developing the rationale and hypothesis based on the findings of degraded speech intelligibility (current lines 128–156).

Previous version

It is unknown, however, that spectral energy distribution is a prominent component of timbre perception. In particular, the first three formants are important in determining both musical timbre in music perception [48] and vowel quality (and thus timbre) in speech perception [49, 50]. A few studies have used speech stimuli and reported F_0-based segregation with vowels [51–55] and syllables [56–58] and formant-based segregation with vowels [52, 59–64]. To our knowledge, no study of stream segregation systematically investigates the combination of F_0 separation and spectral differences among several frequency bands with non-speech stimuli. To elucidate further the combined effects of pitch and timbre on sequential stream segregation, a systematic investigation by varying the spectral energy distribution among several frequency bands is necessary. Specifically, how sequential stream segregation occurs with harmonics dispersed over several frequency bands is largely unknown. Thus, we focused on sequential stream segregation with harmonic complex tones, which were divided into several frequency bands. Following this, harmonics in every other band were removed, and the resulting two possible stripe-like spectral patterns (with either odd-numbered or even-numbered frequency bands, not with odd-numbered or even-numbered harmonics) were alternated with each tone.

Revised version

However, these studies left room open to investigate how timbre differences caused by specific patterns of spectral shapes affect stream segregation. The most recent investigations on degraded speech perception provide clues for extending previous investigations on sequential stream segregation. It has been established that speech intelligibility drastically varies when speech sentences are interrupted in time and frequency [36, 37]. Ueda et al. call such speech *checkerboard speech* because its spectrogram looks like a checkerboard. The intelligibility of checkerboard speech varies drastically depending on the combination of the number of frequency bands and segment duration. The intelligibility of 20- or 16-band checkerboard speech is almost at the ceiling, irrespective of segment duration. However, the intelligibility of two- or four-band checkerboard speech is highest (more than 90%) at 20-ms segment duration, lowest (around 35%–40%) at around 80–160 ms, and moderate (more than 50%) at 320 ms. We may conjecture that spectrotemporal interruption with two or four frequency bands and 80–160-ms segment duration promotes auditory segregation, hampers integration, and reduces intelligibility.

To test this hypothesis, it is necessary to examine how the auditory system integrates or segregates nonspeech stimuli, rapidly switching in time and frequency. Among variables that can be included, we selected fundamental frequency shifts and harmonic number band switching. The fundamental frequency shifts result in pitch shifts, and harmonic number band switching varies timbre. To realize drastic changes in timbre, we group harmonic components into several bands based on harmonic numbers and remove harmonics in every other band. We call these stimuli *stripe tones*. Further-

more, two extreme combinations of pitch and timbre shift directions—a *congruent* shift and an *incongruent* shift—are constructed to capture the whole picture of the possible combinations of these variables. The congruent shift is defined as the combination of upward fundamental frequency shifts and an odd-numbered-band tone to an even-numbered-band tone shift and vice versa, and the incongruent shift is defined as the combination of upward fundamental frequency shifts and an even-numbered-band tone to an odd-numbered-band tone shift and vice versa.

Lines 78–79: A new paragraph started from "We constructed" (current line 157).

Line 79: "these *stripe tone* sequences" was replaced with "*stripe tone sequences*".

Lines 79–81: "to elucidate how the auditory system organizes (groups and segregates) harmonics dispersed over frequency and time (the spectrotemporal domain)" was removed.

Line 81: The following sentences were added (current lines 160–163).

> LHL$_{-}$ tone sequences were combined with congruent shifts in experiment 1 and incongruent shifts in experiment 2 (Fig 2). HLH$_{-}$ tone sequences were combined with congruent shifts in experiment 3 and incongruent shifts in experiment 4 (Fig 3).

Lines 82–92: The whole paragraph was replaced with the following paragraph, providing three research questions and a summary of the findings (current lines 174–184).

Previous version

> Four experiments were conducted to explore how the F_0 separation (a pitch cue) interacted with switching frequency bands (a timbral cue) in sequential stream segregation. To this end, we systematically combined F_0 separation, number of frequency bands, and *congruency* between the shift directions of F_0s and frequency bands (i.e., same and opposite shift directions). The detailed concept of congruency is described in Method (section 2.2). The effect of congruency was examined by comparing the results of experiments 1 and 2, and experiments 3 and 4. The combinations of experiments 1 and 3, and experiments 2 and 4, were to check the effect of the tone sequence patterns, i.e., LHL- and HLH- tone sequences. These experiments revealed the competitive interaction between pitch (by shifting F_0s) and timbre (by switching frequency bands) in sequential stream segregation.

Revised version

> The following three research questions were raised.
>
> 1. Do timbre differences in stripe tones affect the percentages of segregation responses?
> 2. Does congruency in shift directions of pitch and timbre for stripe tone sequences affect percentages of segregation responses?
> 3. Do the stripe tone sequence patterns, LHL$_{-}$ and HLH$_{-}$, interact with the congruency of pitch and timbre shift directions?
>
> With these research questions, we planned to clarify a trade-off between pitch and timbre in sequential stream segregation with stimuli characterized by stripe-like spectral patterns. We found timbre manipulated by the number of bands dominated the results when the number of bands was small (cf. Jhang et al. [38]).

1.4 Comments on the Methods

1. The authors specifically mentioned that some participants attended Technical Listening Training classes (p.5, l.102-108), but they did not discuss how this training might have influenced the results.

   Response We included these descriptions of participants in the previous manuscript, but it is impossible to discuss the effects of training because there was no control group in this respect. Also, the training effects of Technical Listening Training classes have never been examined in formal experiments with control groups. In that sense, the descriptions are excess details. We removed the part and other unnecessary details as well.

   Lines 99–101: Revised as follows (current lines 191–193).

   Previous version

   : The recruitment period for experiments 1–2 started from June 15, 2022 and ended on July 20, 2022; that for experiments 3–4 started from September 12, 2022 and ended on October 8, 2022.

   Revised version

   . The recruitment period for experiments 1 and 2 was from June 15 to July 20, 2022, and for experiments 3 and 4 from September 12 to October 8, 2022.

   Lines 103–105: ", nine of whom had attended the Technical Listening Training classes provided by the Department of Acoustic Design, Faculty of Design, Kyushu University [65]" was removed.

   Lines 105–111: These sentences were moved to Procedure as a new paragraph, which now reads as follows (current lines 349–353), along with the following revisions.

   A group of nine participants, including the participant who had no extracurricular musical training, were assigned to the experiments in the order of 1, 2, 4, and 3. Another group of nine participants were assigned to the experiments in the order of 2, 1, 3, and 4; however, one participant did not participate in experiments 3 and 4. Therefore, the data from the last mentioned participant was excluded from the following analysis.

   Lines 105–106: "(four of them had attended the Technical Listening Training classes)" was removed.

   Lines 107 and 109: "joined" was replaced with "were assigned to".

   Line 108: "eight" was replaced with "nine".

   Line 108: "(six had attended the Technical Listening Training classes)" was removed.

   Lines 109–110: ". One participant finished only experiments 2 and 1 in this order, therefore" was replaced with "; however one participant did not participate in experiments 3 and 4. Therefore".

   Line 110: "of this participant" was replaced with "from the last mentioned participant".

   Line 111: "Thus, 17 participants served in experiments 1–4." was removed.

2. The language background of the participants is missing.

   Response We included the language background of the participants.

Line 98: "students" was replaced with "Japanese-native students" (current line 190).

3. Too much information in 'Conditions'–please add figures to facilitate understanding.

Response We revised Figs 2 and 3 and added Figs 4 and 5 to show the spectrograms of the audio exemplars, control stimuli, and experimental stimuli (stripe tones) separately. We made the following revisions to the Conditions and Stimuli sections.

Lines 115–123: The paragraph was removed.

Lines 124–125 and 134: "one band" or "one-band" were replaced with "full band" or "full-band". Other sections [lines 253 and 428 and Fig 4 (current Fig 7)] were revised in the same manner.

Lines 124–125: Revised as follows (current lines 217–219).

Previous version

Both pure tones and harmonic complex tones with 35 frequency components (one band) were employed in control conditions across the experiments.

Revised version

Both pure tones and (full-band) harmonic complex tones with 35 consecutive harmonics were used as control stimuli (Fig 4) as well as exemplars (Fig 5).

Lines 125–132: Revised as follows (current line 227).

Previous version

Harmonic complex tones with switching frequency bands were used in experimental conditions. These control and experimental conditions were used as test sequences to collect data. In addition, to convey the concepts of one stream (integration) and two streams (segregation), four audio exemplars of stimulus sequences were prepared in each experiment: pure-tone sequences and one-band harmonic complex tone sequences, which were separated by 2-semitone intervals (for one stream; H tones with an F or F_0 of 224.5 Hz) and 18-semitone intervals (for two streams; H tones with an F or F_0 of 565.7 Hz).

Revised version

Stripe tones were used as experimental stimuli.

Line 133: The following sentence was added at the beginning of the paragraph (current lines 228–229).

Exemplars' (fundamental) frequency separations were 2 semitones for "one-stream" exemplars and 18 semitones for "two-stream" exemplars.

Line 134: "stimulus type" was replaced with "control stimulus type".

Line 135: "The range of (fundamental) frequency separations was determined referring to Haywood and Roberts [13–15] and the results of our pilot experiment." was added (current lines 233–234).

Lines 135–137: "Thus, H tones had Fs or F_0s of 252, 356.4, and 504 Hz in test sequences. The LHL- tone sequence pattern was used in experiments 1 and 2, and the HLH- pattern was used in experiments 3 and 4." was removed.

Line 138: "experimental" was replaced with "stripe-tone".

Line 139: "frequency bands" was replaced with "bands". Other sections (lines 241, 265, 266, 267, 272, 362, 383, 413, 416, 424, 426, 428, and 434) were revised in the same manner.

Line 139: "(2, 4, 8, and 16 bands)" was replaced with "(2, 4, 8, and 16 bands in Table 1)".

Lines 140–145: "Regarding the number of frequency bands, it refers to the number of passbands in Tables 1 and 2, except for the one band. As a starting point for investigating the effects of switching frequency bands (2–16 bands) on sequential stream segregation with non-speech stimuli, we used the band settings defined by Ueda et al. [66], which were based on the four essential frequency bands in speech perception [67] and critical bands [68]." was removed.

Tables 1 and 2: The "1 band" column was removed.

Table 1, title: "harmonic complex tone stimuli" was replaced with "L-stripe-tones".

Table 2, title: "the" was removed. "in harmonic complex tone stimuli" was replaced with "for stripe tones".

Lines 146–147 "corresponding harmonic numbers in each passband when the F_0 was 200 Hz, i.e., the L-tone F_0" was replaced with "the correspondence between band numbers and harmonic numbers for stripe tones" (current lines 246–247).

Lines 147–155: The following sentences were removed.

> These sets of harmonic numbers were maintained for all other stimuli with different F_0s to minimize the variations of pitch salience caused by variation in harmonic number (and resolvability), to fix spectral envelope shapes relative to F_0, and to keep the total number of harmonics constant. Every stimulus in the experimental conditions consisted of either odd-numbered bands or even-numbered bands (both of which contained resolved harmonics, except for those of even-numbered two-band stimuli). These two kinds of stimuli alternated (Figs 2 and 3). Thus, the spectral patterns of the stimuli relative to an F_0 moved upward when an even-numbered band stimulus followed an odd-numbered band stimulus, and vice versa.

Lines 157–158: "Figs 2(c)–(f) and 3(c)–(f)" was replaced with "Figs 2(a–h) and 3(a–h)." "Figs 2(i)–(l) and 3(i)–(l)" was replaced with "Figs 2(i–p) and 3(i–p)."

Lines 158–163: The following sentences were removed.

> Note that "incongruent" does not mean that the spectral patterns of L tones always occupy higher frequency regions than those of H tones in a tone sequence [e.g., Figs 2(l) and 3(l)] because the occupied frequency regions depend on the combinations of F_0s and frequency bands (with corresponding harmonic numbers relative to F_0). Congruent stimuli were used in experiments 1 and 3, and incongruent stimuli were used in experiments 2 and 4.

Lines 163–164: Revised as follows (current lines 265–266).

Previous version

> The full sets of stimulus spectrograms are provided in the Supporting information (S1–S6 Figs).

Revised version

> For the full set of stimulus spectrograms, see S1–S6 Figs.

Line 164: The following sentences were added (current lines 266–270).

> For each experiment, six control conditions [three steps of (fundamental) frequency separations × two control stimulus types] and 12 stripe-tone

conditions (three steps of fundamental frequency separations × four steps of the number of bands) were constructed. Thus, 18 conditions were prepared for each experiment.

Fig 2: (b) was moved to new Fig 4 as (a). (a), (g) and (h) were removed.

Fig 3: (b) was moved to new Fig 4 as (d). (a), (g) and (h) were removed.

New Fig 4, title: Spectrograms of control stimuli with (full-band) harmonic complex tones.

New Fig 4, caption: LHL_ sequences with (a) 4-, (b) 10-, and (c) 16-semitone separation were used in both experiments 1 and 2. HLH_ sequences with (d) 4-, (e) 10-, and (f) 16-semitone separation were used in both experiments 3 and 4. Pure-tone stimuli were also used but are not shown here.

New Fig 5, title: Spectrograms of audio exemplars with (full-band) harmonic complex tones.

New Fig 5, caption: (a) The LHL_ audio exemplar for one stream (integration) with 2-semitone separation and (b) the LHL_ audio exemplar for two streams (segregation) with 18-semitone separation were used in both experiments 1 and 2. HLH_ exemplars for one stream and two streams (c and d) with 2- and 18-semitone separation were used in both experiments 3 and 4. Note that the 2-semitone separation, as in Fig 5(a and c), was narrower than the 4-semitone separation, which was the narrowest (fundamental) frequency separation used in measuring segregation. Likewise, the 18-semitone separation, as in Fig 5(b and d), was wider than the 16-semitone separation, which was the widest (fundamental) frequency separation used in measuring segregation. Pure-tone audio exemplars were also used (not shown here).

Fig 2, title: Replaced as follows. "Spectrograms of stripe tones with LHL_ triplets."

Fig 2, caption: The whole caption was replaced with the following.

Stripe tones are harmonic complex tones divided into 2, 4, 8, or 16 bands. Frequency components are grouped into several bands based on harmonic numbers (Tables 1 and 2), and the components in every other band are removed. The tone duration is 80 ms. Two possible spectral patterns (with only odd- or even-numbered bands) are switched between L and H tones to make a sequence. An LHL_ ("_" denotes a "silent gap") triplet is presented three times with 80-ms gaps. The columns are in the order of 2-, 4-, 8-, and 16-band stimuli from left to right. The upper two rows (a–h) represent congruent sequences used in experiment 1 (fundamental frequencies and spectral patterns move in the same direction), and the lower two rows (i–p) represent incongruent sequences used in experiment 2 (fundamental frequencies and spectral patterns move in the opposite direction). The first and third rows from the top (a–d and i–l) represent stimuli with a four-semitone fundamental frequency separation. The second and fourth rows (e–h and m–p) represent stimuli with a 16-semitone separation. The L-tone fundamental frequency is fixed at 200 Hz. The H-tone fundamental frequency for the four-semitone separation is 252 Hz, and the 16-semitone separation is 504 Hz.

Fig 3, title: Replaced as follows. "Spectrograms of stripe tones with HLH_ triplets."

Fig 3, caption: The whole caption was replaced with the following.

Congruent and incongruent sequences (a–h and i–p) were used in experiments 3 and 4. The columns are in the order of 2-, 4-, 8-, and 16-band stimuli from left to right. The first and third rows (a–d and i–l) represent stimuli with a four-semitone separation, and the second and fourth rows (e–h and m–p) represent stimuli with a 16-semitone separation.

Figs 2 and 3: Redrawn for clarity. S2–S6 Figs were revised similarly.

(a) The frequency axis was plotted on a log scale.

(b) The first and the last markers on the time axis were moved to the onset of the first triplet and the offset of the final triplet.

(c) The four values on the time axis were replaced with "0, 320, 640, 880".

(d) "Time (s)" was replaced with "Time (ms)".

(e) The numbers or words on each panel were removed, except for those in the leftmost column and the lowest row.

(f) A label of 2 bands, 4 bands, 8 bands, and 16 bands was added for each column from left to right.

Line 166: A paragraph was added at the beginning of the Stimuli section. The sentences in lines 166–167 and 172–174 were moved to the second and first sentences in the new paragraph. Thus, the whole paragraph reads as follows (current lines 272–278).

> All stimuli were generated with custom software written in J language [41], with a sampling frequency of 44100 Hz and a quantization of 16 bits. Harmonic complex tone stimuli were generated by adding equal-amplitude components in sine phase. L (fundamental) frequency was fixed at 200 Hz. H (fundamental) frequencies for 2-, 4-, 10-, 16-, and 18-semitone separations were 225, 252, 356, 504, and 566 Hz. The stimulus and gap duration was 80 ms, including 10-ms rise and fall time with raised-cosine amplitude envelopes for a stimulus.

Line 167: "each tone" was replaced with "each stimulus".

Line 168: The following sentences were added after "calibration tone." (current lines 282–286).

> The sound pressure level (SPL) of the calibration tone was adjusted to 70 dB (A). The SPLs at headphone outputs were measured with an artificial ear (Brüel & Kjær type 4153, Brüel & Kjær Sound & Vibration Measurement A/S, Nærum, Denmark), a condenser microphone (Brüel & Kjær type 4192), and a sound level meter (Brüel & Kjær type 2260).

Lines 168–171: Revised as follows (current lines 290–291)

Previous version

> Further adjustments to equalize the SPLs of the tones were done by monitoring headphone outputs (we made 60-second tones for monitoring headphone outputs, and applied the adjustments for the tones of 80-ms duration). The level-adjusted tones were ordered and combined with gaps to construct the stimulus sequences.

Revised version

> Each stimulus SPL was confirmed with a 60-second tone of the same frequency composition.

4. The variables from control conditions and experimental conditions seem incomparable. The 'stimuli type' in control condition and 'number of frequency bands' in experimental condition appear to refer to the same thing–the authors should consider making their names consistent. This issue occurs throughout the manuscript.

Response No, the "stimulus types" for control stimuli and the "number of bands" of stripe tones are different. We revised the Conditions to clarify this point by replacing "stimulus types" with "control stimulus types" and "experimental conditions" with

"stripe-tone conditions". Furthermore, to avoid confusion, the terms "number of frequency bands" and "frequency bands" for the stripe tones with fixed harmonic number groups were replaced with "number of bands" and "bands" throughout the manuscript.

Lines 134 and 237: "stimulus types" was replaced with "control stimulus types".

Lines 138, 232, 240, 263, and 264 and the titles of Fig 5 (current Fig 8) and S2 Table: "experimental conditions" was replaced with "stripe-tone conditions".

Lines 139, 241, 265, 266, 267, 272, 362, 383, 413, 416, 424, 426, 428, and 434: "frequency bands" was replaced with "bands".

5. The procedure section is lengthy, disorganized, and not reader-friendly. Some information overlaps with details about the stimuli (e.g., p.10, l.183-186). Please explain the procedure briefly and clearly. Consider adding a flowchart and a summary.

Response The Procedure was streamlined with the following revisions.

Line 176: ", including instructions, practice trial blocks, and main trial blocks," was removed.

Line 177: "sound-attenuated" was replaced with "sound-attenuating".

Lines 183–186: The descriptions about sound pressure measurement procedures were moved to the Stimuli and revised as follows (current lines 282–286).

> The SPLs at headphone outputs were measured with an artificial ear (Brüel & Kjær type 4153, Brüel & Kjær Sound & Vibration Measurement A/S, Nærum, Denmark), a condenser microphone (Brüel & Kjær type 4192), and a sound level meter (Brüel & Kjær type 2260).

Lines 187–193: Revised as follows (current lines 312–314).

Previous version

> In the instruction phase, the concepts of one stream and two streams were conveyed through written instructions with schematic illustrations. Also, audio exemplars that demonstrated clear one-stream and two-stream percepts were presented, following the procedure by Haywood and Roberts [13–15], Grimault et al. [20, 26], and Thomassen et al. [7]. The F and F_0 separations of one-stream exemplars (2 semitones) and two-stream exemplars (18 semitones) were outside the range of those in test sequences (4–16 semitones), therefore, participants had to generalize the concept in the experiment.

Revised version

> The experimenter explained the concepts of one stream and two streams to the participants using schematic illustrations (Fig 6), written explanations, and audio exemplars.

New Fig 6: A new figure showing the schematic illustrations of the streaming concept was included.

Title: Schematic illustrations given to participants.

Caption: Schematic illustrations for an $LHL\_$ sequence were used in experiments 1 and 2: (a) a test sequence, (b) perception of one stream, and (c) perception of two streams. Similar schematic illustrations were used for an $HLH\_$ sequence in experiments 3 and 4 (d–f). The dashed lines represent perceptual connections between tones. Participants were instructed to focus on the final triplet (marked with squares) and report whether they heard one stream or two streams. Reproduced with translations from Japanese to English.

Line 196: "(LHL- in experiments 1 and 2; HLH- in experiments 3 and 4) and instructed that we call the percept" was replaced with "called".

Lines 196–198: We elaborated on how the exemplars were presented (current lines 319–323).

Previous version

> Then, two one-stream exemplars were presented in the order of pure-tone sequence and one-band harmonic complex tone sequence. A similar procedure was carried out for the two-stream exemplars.

Revised version

> Then, pure and full-band tone exemplars with two-semitone separation were presented in this order. Subsequently, they were told that they would hear exemplars sound like a distinctly segregated sequence called "two streams" and presented with pure and full-band tone exemplars with 18-semitone separation.

Line 199: "another" was replaced with "presentations to be repeated".

Lines 199–208: These sentences were moved to just after line 217 as a new paragraph, which now reads as follows (current lines 344–348), along with the following revisions.

> Each trial started after a three-second pause to eliminate the build-up effect on stream segregation from previous trials. Each trial block consisted of 18 trials of conditions that were randomly ordered. After two practice trial blocks, 20 main trial blocks were run. Thus, each condition was measured 20 times. Participants were allowed to take a break between the blocks.

Lines 199-201: "On each trial (in the phases of practice trial blocks and main trial blocks), the test sequence was presented once" was replaced with "Each trial started".

Lines 202–205: "The 18 test sequences were randomly ordered in each trial block: six combinations in control conditions (three steps of F_0 separations $\times$ two stimulus types) and 12 combinations in experimental conditions (three steps of F_0 separations $\times$ four steps of number of frequency bands)." was replaced with "Each trial block consisted of 18 trials of conditions that were randomly ordered."

Line 205: "A total of" was replaced with "After two practice trial blocks,"

Line 205: "were run" was added after "trial blocks".

Lines 205–206: "(each combination" was replaced with "Thus, each condition".

Lines 205–206: ") started after presenting two practice trial blocks" was removed.

Lines 207–208: "Practice trial blocks were prepared so that participants could be familiarized with the task." was removed.

Lines 209–210: "(i.e., the last three tones)" was removed.

Line 210: "three triplet sequences" was replaced with "three-triplet sequences".

Line 211: "two streams" was replaced with "two streams (Fig 6)".

Line 213: ", following the instructions by Haywood and Roberts [13–15]" was removed.

Lines 214–215: Revised as follows (current lines 340–341).

Previous version

> to click either the button "One" or "Two" on a computer screen to indicate a response of either one stream or two streams.

Revised version

to select a "One" or "Two" response button on a computer screen.

Lines 216–217: Revised as follows (current line 343).

Previous version

and participants were allowed to cancel and re-enter their responses.

Revised version

providing an opportunity to correct an erroneous input.

Lines 219–220: Revised as follows (current lines 355–356).

Previous version

and took about 60 to 100 minutes for completing each experiment.

Revised version

, taking 60 to 100 minutes to complete each one.

Lines 222–227: The following details were removed.

Three participants were asked to join experiments 1 and 2 again at least 19 days later (before they joined experiments 3 and 4) because we had to fix an issue in the computer program: When a participant double-clicked the response buttons "One" or "Two," a message box appeared twice. However, sometimes two responses were recorded without presenting the second trial. The previous data for these participants were discarded and replaced with the data obtained with the refined program.

6. P.10, l.191-193: Does this mean that they learned the concept during instruction using stimuli that were not used in the experiment, and then had to apply that concept on their own during the experiment? If so, it seems similar to standard instructions used in many perception experiments. Since the authors mentioned it specifically and in great detail, I'm wondering if there is a particular reason for that.

Response The following details in lines 187–193 were removed.

In the instruction phase, the concepts of one stream and two streams were conveyed through written instructions with schematic illustrations. Also, audio exemplars that demonstrated clear one-stream and two-stream percepts were presented, following the procedure by Haywood and Roberts [13–15], Grimault et al. [20, 26], and Thomassen et al. [7]. The F and F_0 separations of one-stream exemplars (2 semitones) and two-stream exemplars (18 semitones) were outside the range of those in test sequences (4–16 semitones), therefore, participants had to generalize the concept in the experiment.

7. P.11, l.215-216: For participants who cancelled and re-entered their responses, which response did the authors use for the final analysis–the first one or the re-entered one? Were they allowed to hear the stimuli a second time? Why did they set up a confirmation box here?

Response This is just to confirm the input. The last part of the sentence, "and participants were allowed to cancel and re-enter their responses." was replaced with "providing an opportunity to correct an erroneous input."

1.5 Comments on the Results

For experimental condition, since the model revealed significant two way and three-way interaction, I am wondering if the authors conducted any post-hoc analysis?

Response Yes, we had conducted it. We reported Tukey's honestly significant difference (HSD) tests in the following revisions.

Line 232: "experimental" was replaced with "stripe-tone".

Lines 233–235: "All combinations of independent variables were included in the model to avoid removing the most powerful combinations from the model, even though some combinations might cause extremely high portions of one- or two-stream responses." was removed.

Lines 236–238: We noticed that "congruency" should be treated as one of the random variables in the analysis for the control condition's data. Therefore, we removed "congruency" from the list of fixed effects. Together with other revisions, the list of the fixed effects was revised as "(fundamental) frequency separation, control stimulus type, tone sequence pattern (all categorical predictors), and their interactions." (current lines 374–375).

Lines 238–240: "Congruency was included in the analysis to see the contextual effect of the combinations with the experimental stimuli, although the control stimuli themselves were irrelevant to congruency." was removed.

Line 240: The following explanation for the analysis model and the list of random effects were included (current lines 378–383).

> The analysis model with these fixed effects was fitted to the data with the following candidate sets of random effects: (1) congruency and participant, (2) congruency and extracurricular musical training nested under participant, (3) congruency and trial block (i.e., block number 1–20 in each experiment) nested under participant, and (4) congruency, trial block nested under participant, and experiment order.

Line 240: A new paragraph started from "Regarding" (current line 384).

Lines 243–245: We revised the analysis with more combinations of random effects. Therefore, the part was revised as follows (current lines 389–393).

Previous version

> With these fixed effects, both the models of control and experimental conditions were fitted with the following candidates of random effects: (1) participant and (2) block number (i.e., block number 1–20 in each experiment) nested under participant.

Revised version

> The analysis model with these fixed effects was fitted to the data with the following candidate sets of random effects: (1) participant, (2) extracurricular musical training nested under participant, (3) trial block nested under participant, and (4) trial block nested under participant and experiment order.

Line 249: The following descriptions about power analysis and multiple comparisons were included (current lines 397–399).

> The statistical power of the fixed effects was estimated with JMP Pro [43] based on 1000-times simulation at an alpha level of 0.05. Post-hoc multiple comparisons with Tukey's honestly significant difference (HSD) tests were conducted.

Lines 253–254: The improved statistical analysis affected the descriptions of the results in control stimulus conditions (current lines 403–408).

Previous version

For the control conditions, the percentages of segregation responses for pure-tone stimuli and one-band harmonic complex tone stimuli increased with widening F or F_0 separation.

Revised version

The percentages of segregation responses for pure and full-band harmonic complex tones increased with widening (fundamental) frequency separations. Full-band harmonic complex tones showed higher percentages of segregation responses than pure tones except for the four-semitone separation. The effect of tone-sequence patterns was negligible.

Lines 255–262: The report of statistical analysis was replaced as follows (current lines 416–427).

These observations were supported by GLMM analysis. A model with random effects of congruency and trial block nested under participant was selected, based on the smallest indices ($-2ResidualLogPseudo\text{-}Likelihood = 56803.2$; generalized $\chi^2/df = 1.67$). The model revealed fixed effects of (fundamental) frequency separation [$F(2,8148) = 689.64$, $p < 0.001$, statistical power $= 0.998$], control stimulus types [$F(1,8148) = 44.92$, $p < 0.001$, statistical power $= 0.886$], and their interaction [$F(2,8148) = 6.33$, $p = 0.002$, statistical power $= 0.731$]. No other fixed effects reached p levels < 0.05 (S1 Table). Multiple comparisons with Tukey's HSD tests revealed that full-band harmonic complex tones showed greater percentages of segregation responses than pure tones at 10-semitone separation ($t = -18.49$, $p < 0.001$) and 16-semitone separation ($t = -15.21$, $p < 0.001$), but not at 4-semitone separation ($t = 0.6$, $p > 0.999$).

Lines 282–291: The report of statistical analysis was replaced as follows (current lines 459–480).

These observations were supported by GLMM analysis. A model with random effects of trial block nested under participant and experiment order was selected, based on $-2ResidualLogPseudo\text{-}Likelihood = 132565.59$ and generalized $\chi^2/df = 0.67$. The model revealed the two-way interaction effect of number of bands $\times$ fundamental frequency separation [$F(6,16272) = 9.6$, $p < 0.001$, statistical power $= 0.874$] and the three-way interaction effect of congruency $\times$ number of bands $\times$ fundamental frequency separation [$F(6,16272) = 22.52$, $p < 0.001$, statistical power $= 0.926$]. No other fixed effects reached p levels < 0.05 (S2 Table). Tukey's HSD tests revealed that congruent 8-band stimuli were more frequently judged to be segregated than incongruent 8-band stimuli at 4-semitone ($t = 11.99$, $p < 0.001$) and 10-semitone separation ($t = 12.51$, $p < 0.001$). By contrast, with 16 bands, incongruent stimuli were more frequently judged to be segregated than congruent stimuli at 10-semitone ($t = -7.88$, $p < 0.001$) and 16-semitone separation ($t = -6.84$, $p < 0.001$). However, congruent 16-band stimuli may be more frequently judged to be segregated at 4-semitone separation ($t = 3.66$, $p = 0.046$). All other comparisons at 4-, 10-, and 16-semitone separations between the corresponding conditions concerning congruency and number of bands resulted in p levels exceeding 0.05. Moreover, incongruent 8-band stimuli were more frequently judged to be segregated than incongruent 16-band stimuli at the 4-semitone separation ($t = 13.78$, $p < 0.001$); however, incongruent 16-band stimuli were

more frequently judged to be segregated than incongruent 8-band stimuli at 10-semitone ($t = -13.19$, $p < 0.001$) and 16-semitone separations ($t = -9.02$, $p < 0.001$).

S1 and S2 Tables: The GLMM analysis summaries for the control and stripe-tone conditions were revised according to the newly performed statistical analyses.

1.6 Comments on the Discussions

1. I am not clear about the reasoning for keeping section 4.1 in the discussion–it sounds more like a justification of the methods, which would be more appropriate in the methods section.

   Response We believe some descriptions in section 4.1.1 should be included in the Discussion section because replication not only supports the method as appropriate but also proves the experiments were conducted correctly and reliably. However, we made an effort to shorten section 4.1 by shortening section 4.1.1. We removed sections 4.1.2 and most of 4.1.3 because the arguments are nonessential. We revised section 4.1 as follows.

   Line 293: The section title was revised as "Replications".

   Lines 295–359: Revised as follows (current lines 549–554).

   > A larger (fundamental) frequency separation, thus a greater pitch difference, caused more frequent segregation responses for both pure-tone and full-band stimuli. Additionally, full-band stimuli with many harmonic components tended to be more segregated than pure-tone stimuli. Rajasingam et al. [19] and Roberts and Haywood [21] found that two-component complex tones were perceived to be more segregated than pure tones. The current results seem to be in line with those of previous studies.

2. The title of 4.2 is simply a collection of words rather than a summary of content.

   Response The section title was revised as "Pitch and timbre trade-off in stream segregation".

3. Half of section 4.2 repeats the results rather than discussing them.

   Response Sections 4.2 and 4.3 were combined into one section, in which the research questions posed in the Introduction were considered. The section was revised as follows.

   Lines 361–375: The whole paragraph was revised as follows (current lines 557–560) and connected to the next paragraph.

   > The interaction effect of fundamental frequency separation, number of bands, and congruency was observed for stripe-tone conditions.

   Lines 376–378: Revised as follows (current lines 575–576).

   Previous version

   > In other words, a trade-off between pitch (by shifting F_0s) and timbre (by switching frequency bands) on sequential stream segregation, and therefore auditory grouping, was observed in the current results.

   Revised version

   > This means that the pitch and timbre trade-off on sequential stream segregation was observed overall.

Line 379: “occurred” was replaced with “was observed”.

Line 380: “does capture” was replaced with “captures”.

Line 381: “This also means that the auditory system has difficulty in” was replaced with “However, the auditory system has difficulty”.

Lines 383–384: “closer to stimuli with a continuous spectrum (i.e., a single band)” was replaced with “closer to full-band stimuli”.

Lines 384–385: “The results of the LHL- and HLH- sequences consistently support the claims.” was removed.

Line 386: The section title for 4.3 was removed, and the text from line 391 to line 398 was merged into the previous section (current lines 589–596).

Lines 388–391: “We extended the previous findings about competition between pitch and timbre in sequential stream segregation by changing F_0 separations of complex tones with rich harmonics and contrasting blocks of harmonics dispersed over the spectrotemporal domain (with 2–16 frequency bands).” was removed.

Line 391: “competition” was replaced with “a trade-off”.

Lines 396–398: Revised as follows (current lines 596–599).

Previous version

A transition from a segregated percept to an integrated percept was realized by increasing the number of frequency bands for the stimuli.

Revised version

Manipulating the number of bands was reflected in the percentages of segregation responses. Thus, the first research question, “Do timbre differences in stripe tones affect the percentages of segregation responses?” was answered with “Yes.”

4. I don’t see the purpose of introducing the spectral centroid starting from p18, l. 399. It seems like the authors are introducing new concepts and results in the discussion, making the study feel somewhat ad hoc and overly exploratory.

Response Lines 399–405 and Fig 6 were removed. Instead, the answers to the second and third research questions, as well as the discussion about our recently published paper, are presented here in three new paragraphs as follows (current lines 610–644).

The answer to the second research question, “Does congruency in shift directions of pitch and timbre for stripe tone sequences affect percentages of segregation responses?” is that “It depends on the number of bands.” No effect of congruency was observed for two- and four-band stimuli: These stimuli were always perceived to be segregated, irrespective of the sizes of pitch separations and congruency. Listeners perceived congruent eight-band stimuli as segregated most of the time. However, they reported segregation less frequently for incongruent eight-band stimuli. In contrast, their proportions of segregation responses varied mainly according to the sizes of pitch separations for 16-band stimuli: Segregation responses increased as pitch separations became greater. Proportions of segregation responses became greater for incongruent stimuli than for congruent stimuli with 10 or 16 semitone separations, very probably due to the absence of any frequency components in the lowest band of L tones for the congruent 16-band stimuli [Table 2 and Fig 2(h)]. Still, the differences due to congruency were slight. Thus, when the number of bands is two or four, prominent timbre differences between odd- and even-numbered-band stripe tones should

mainly govern perceptual segregation. When the number of bands becomes eight or more, timbre differences between odd- and even-numbered-band stripe tones become less competitive with pitch differences, leading to the segregation responses being more governed by pitch differences.

During the revision process of the current paper, we were acknowledged that our another paper on sequential stream segregation by using *band tones* was accepted for publication [38]. Band tones are harmonic complex tones that consist of odd- or even-numbered bands with fixed passbands (comparable to Table 1), unlike the fixed harmonic number groups used for stripe tones (Table 2). With band tones, listeners perceived eight-band tones as segregated most of the time, except for the congruent four-semitone separation. Moreover, with eight bands, segregation responses were reported more often for band tones than for stripe tones. Thus, the current results indicated that the trade-off between pitch and timbre on stream segregation appeared with a sharper contrast with stripe tones than band tones, suggesting that the effects of pitch and timbre differences on perceptual segregation and integration are somewhat balanced around eight-band stimuli.

The answer to the third question, "Do the stripe tone sequence patterns, LHL$_{-}$ and HLH$_{-}$, interact with the congruency of pitch and timbre shift directions?" was "No." Tone sequence patterns had practically no effect on segregation, replicating previous results by Thomassen et al. [20] with pure tones.

5. I noticed that musical training background wasn't controlled for or considered as a predictive factor in this study, yet the authors suddenly introduce it (starting from l. 406) and spend quite a few lines expanding on this point.

Response We removed lines 406–408. In lines 240 and 243–245, we included "extracurricular musical training" as a candidate random effect in our GLMM analysis (current lines 378–383 and 389–393). Still, it turned out that introducing that effect did not improve model fitting.

Lines 419–420: Replaced as follows (current lines 659–660).

Previous version

Further investigations are warranted to clarify how participant musical training affects the results.

Revised version

Obviously, the auditory system shows limits in integrating spectrotemporally scattered harmonics.

1.7 Comments on the Conclusions

Please state the theoretical significance and potential social contribution of the study.

Response The sentence in lines 435–436 was revised as follows (current lines 677–678).

Previous version

Further investigations are warranted to clarify how the way of switching frequency bands and the prolonged sequences affect the results.

Revised version

The current investigation formed a basis for further investigations on detecting an auditory target in a noisy background.

2 Reply to Reviewer 2

2.1 General Comments

The manuscript presents an interesting study investigating the interaction between fundamental frequency (F0) separation and switching frequency bands on auditory stream segregation. The design of the four experiments is good, and the experiments were properly executed. However, it could benefit from clearer justifications for some methodological choices, e.g., the specific range of F0 separations and the exact harmonic numbers chosen for stimuli. Also, the number of participants is relatively small, and their experience of musical/listening training is also diverse. This can significantly influence the results of the experiments. My major concern over this paper is the number of participants (17 only). I strongly recommend the authors to increase the number or to present a power analysis of the results. Also, the influence of gender and age has not been fully illustrated.

The data supports the conclusions, i.e., stream segregation depends more on timbral contrast when fewer frequency bands are used, whereas F0 separation dominates for higher numbers of bands. I would suggest a more detailed discussion on the practical significance of the findings. There is some discussion between Line 413 and 419, which could be further expanded.

A minor comment is that the writing style can be improved by making some sentences more academic and the methodology part more concise.

Response We are pleased to note your favorable opening remarks. Thank you very much for providing helpful suggestions, which can be summarized into the following four points.

1. Justifications of methodological choices should be presented.

   Response We made the following revision. As to the harmonic numbers, please refer to the caption of Table 2.

   Line 135: The reason that we chose the range of (fundamental) frequency separations was indicated as "The range of (fundamental) frequency separations was determined referring to Haywood and Roberts [13–15] and the results of our pilot experiment." (current lines 233–234).

2. Statistical power should be presented, and the influence of training, gender, and age has not been fully illustrated.

   Response Power analyses of the results were reported. The effect of extracurricular musical training was considered as a part of candidate random effects in GLMM fitting. However, there is no reason to believe that gender and age affect stream segregation. Moreover, we fitted the model with the random effects of age and gender, but it turned out that introducing these effects did not improve model fitting. Therefore, we decided not to describe these two effects in the manuscript. The descriptions of Technical Listening Training classes were removed because the training effects have never been verified with formal experiments.

Lines 103–105: ", nine of whom had attended the Technical Listening Training classes provided by the Department of Acoustic Design, Faculty of Design, Kyushu University [65]" was removed.

Lines 105–111: These sentences were moved to Procedure as a new paragraph, which now reads as follows (current lines 349–353), along with the following revisions.

> A group of nine participants, including the participant who had no extracurricular musical training, were assigned to the experiments in the order of 1, 2, 4, and 3. Another group of nine participants were assigned to the experiments in the order of 2, 1, 3, and 4; however, one participant did not participate in experiments 3 and 4. Therefore, the data from the last mentioned participant was excluded from the following analysis.

- **Lines 105–106:** "(four of them had attended the Technical Listening Training classes)" was removed.
- **Lines 107 and 109:** "joined" was replaced with "were assigned to".
- **Line 108:** "eight" was replaced with "nine".
- **Line 108:** "(six had attended the Technical Listening Training classes)" was removed.
- **Lines 109–110:** ". One participant finished only experiments 2 and 1 in this order, therefore" was replaced with "; however one participant did not participate in experiments 3 and 4. Therefore".
- **Line 110:** "of this participant" was replaced with "from the last mentioned participant".
- **Line 111:** "Thus, 17 participants served in experiments 1–4." was removed.

Lines 236–238: We noticed that "congruency" should be treated as one of the random variables in the analysis for the control condition's data. Therefore, we removed "congruency" from the list of fixed effects. Together with other revisions, the list of the fixed effects was revised as "(fundamental) frequency separation, control stimulus type, tone sequence pattern (all categorical predictors), and their interactions." (current lines 374–375).

Lines 238–240: "Congruency was included in the analysis to see the contextual effect of the combinations with the experimental stimuli, although the control stimuli themselves were irrelevant to congruency." was removed.

Line 240: The following explanation for the analysis model and the list of random effects were included (current lines 378–383).

> The analysis model with these fixed effects was fitted to the data with the following candidate sets of random effects: (1) congruency and participant, (2) congruency and extracurricular musical training nested under participant, (3) congruency and trial block (i.e., block number 1–20 in each experiment) nested under participant, and (4) congruency, trial block nested under participant, and experiment order.

Line 240: A new paragraph started from "Regarding" (current line 384).

Lines 243–245: We revised the analysis with more combinations of random effects. Therefore, the part was revised as follows (current lines 389–393).

Previous version

> With these fixed effects, both the models of control and experimental conditions were fitted with the following candidates of random effects: (1) participant and (2) block number (i.e., block number 1–20 in each experiment) nested under participant.

Revised version

> The analysis model with these fixed effects was fitted to the data with the following candidate sets of random effects: (1) participant, (2) extracurricular musical training nested under participant, (3) trial block nested under participant, and (4) trial block nested under participant and experiment order.

Line 249: The following descriptions about power analysis and multiple comparisons were included (current lines 397–399).

> The statistical power of the fixed effects was estimated with JMP Pro [43] based on 1000-times simulation at an alpha level of 0.05. Post-hoc multiple comparisons with Tukey's honestly significant difference (HSD) tests were conducted.

Lines 253–254: The improved statistical analysis affected the descriptions of the results in control stimulus conditions (current lines 403–408).

Previous version

> For the control conditions, the percentages of segregation responses for pure-tone stimuli and one-band harmonic complex tone stimuli increased with widening F or F_0 separation.

Revised version

> The percentages of segregation responses for pure and full-band harmonic complex tones increased with widening (fundamental) frequency separations. Full-band harmonic complex tones showed higher percentages of segregation responses than pure tones except for the four-semitone separation. The effect of tone-sequence patterns was negligible.

Lines 255–262: The report of statistical analysis was replaced as follows (current lines 416–427).

> These observations were supported by GLMM analysis. A model with random effects of congruency and trial block nested under participant was selected, based on the smallest indices ($-2ResidualLogPseudo\text{-}Likelihood =$ 56803.2; generalized $\chi^2/df = 1.67$). The model revealed fixed effects of (fundamental) frequency separation [$F(2, 8148) = 689.64$, $p < 0.001$, statistical power $= 0.998$], control stimulus types [$F(1, 8148) = 44.92$, $p <$ 0.001, statistical power $= 0.886$], and their interaction [$F(2, 8148) = 6.33$, $p = 0.002$, statistical power $= 0.731$]. No other fixed effects reached p levels < 0.05 (S1 Table). Multiple comparisons with Tukey's HSD tests revealed that full-band harmonic complex tones showed greater percentages of segregation responses than pure tones at 10-semitone separation ($t = -18.49$, $p < 0.001$) and 16-semitone separation ($t = -15.21$, $p < 0.001$), but not at 4-semitone separation ($t = 0.6$, $p > 0.999$).

Lines 282–291: The report of statistical analysis was replaced as follows (current lines 459–480).

> These observations were supported by GLMM analysis. A model with random effects of trial block nested under participant and experiment order was selected, based on $-2ResidualLogPseudo\text{-}Likelihood = 132565.59$ and generalized $\chi^2/df = 0.67$. The model revealed the two-way interaction effect of number of bands $\times$ fundamental frequency separation [$F(6, 16272) =$ 9.6, $p < 0.001$, statistical power $= 0.874$] and the three-way interaction effect of congruency $\times$ number of bands $\times$ fundamental frequency separation [$F(6, 16272) = 22.52$, $p < 0.001$, statistical power $= 0.926$]. No

other fixed effects reached p levels < 0.05 (S2 Table). Tukey's HSD tests revealed that congruent 8-band stimuli were more frequently judged to be segregated than incongruent 8-band stimuli at 4-semitone ($t = 11.99$, $p < 0.001$) and 10-semitone separation ($t = 12.51$, $p < 0.001$). By contrast, with 16 bands, incongruent stimuli were more frequently judged to be segregated than congruent stimuli at 10-semitone ($t = -7.88$, $p < 0.001$) and 16-semitone separation ($t = -6.84$, $p < 0.001$). However, congruent 16-band stimuli may be more frequently judged to be segregated at 4-semitone separation ($t = 3.66$, $p = 0.046$). All other comparisons at 4-, 10-, and 16-semitone separations between the corresponding conditions concerning congruency and number of bands resulted in p levels exceeding 0.05. Moreover, incongruent 8-band stimuli were more frequently judged to be segregated than incongruent 16-band stimuli at the 4-semitone separation ($t = 13.78$, $p < 0.001$); however, incongruent 16-band stimuli were more frequently judged to be segregated than incongruent 8-band stimuli at 10-semitone ($t = -13.19$, $p < 0.001$) and 16-semitone separations ($t = -9.02$, $p < 0.001$).

S1 and S2 Tables: The GLMM analysis summaries for the control and stripe-tone conditions were revised according to the newly performed statistical analyses.

3. A more detailed discussion on the practical significance of the findings is suggested.

Response This investigation tested the auditory system functions in extreme cases; therefore, the practical significance of this is still unknown. However, we may present some possibility of generalizing the findings in line 419 as follows (current lines 659–660).

> Obviously, the auditory system shows limits in integrating spectrotemporally scattered harmonics.

Additionally, we added the following sentence at the end of the Conclusions (current lines 677–678).

> The current investigation formed a basis for further investigations on detecting an auditory target in a noisy background.

4. Writing style can be improved.

Response Thank you for the suggestions. The Method was streamlined with the following revisions. Other revisions, including those in the Results, were made to improve the writing style.

(a) The descriptions in the Participants section were polished with the following revisions.

Line 98: "students" was replaced with "Japanese-native students" (current line 190).

Lines 99–101: Revised as follows (current lines 191–193).

Previous version

: The recruitment period for experiments 1–2 started from June 15, 2022 and ended on July 20, 2022; that for experiments 3–4 started from September 12, 2022 and ended on October 8, 2022.

Revised version

. The recruitment period for experiments 1 and 2 was from June 15 to July 20, 2022, and for experiments 3 and 4 from September 12 to October 8, 2022.

Lines 103–105: “, nine of whom had attended the Technical Listening Training classes provided by the Department of Acoustic Design, Faculty of Design, Kyushu University [65]” was removed.

Lines 105–111: These sentences were moved to Procedure as a new paragraph, which now reads as follows (current lines 349–353), along with the following revisions.

> A group of nine participants, including the participant who had no extracurricular musical training, were assigned to the experiments in the order of 1, 2, 4, and 3. Another group of nine participants were assigned to the experiments in the order of 2, 1, 3, and 4; however, one participant did not participate in experiments 3 and 4. Therefore, the data from the last mentioned participant was excluded from the following analysis.

- **Lines 105–106:** “(four of them had attended the Technical Listening Training classes)” was removed.
- **Lines 107 and 109:** “joined” was replaced with “were assigned to”.
- **Line 108:** “eight” was replaced with “nine”.
- **Line 108:** “(six had attended the Technical Listening Training classes)” was removed.
- **Lines 109–110:** “. One participant finished only experiments 2 and 1 in this order, therefore” was replaced with “; however one participant did not participate in experiments 3 and 4. Therefore”.
- **Line 110:** “of this participant” was replaced with “from the last mentioned participant”.
- **Line 111:** “Thus, 17 participants served in experiments 1–4.” was removed.

(b) The Conditions and Stimuli sections were reorganized with the following revisions. Furthermore, we revised Figs 2 and 3 and added Figs 4 and 5 to show the spectrograms of the audio exemplars, control stimuli, and experimental stimuli (stripe tones) separately.

Lines 115–123: The paragraph was removed.

Lines 124–125 and 134: “one band” or “one-band” were replaced with “full band” or “full-band”. Other sections [lines 253 and 428 and Fig 4 (current Fig 7)] were revised in the same manner.

Lines 124–125: Revised as follows (current lines 217–219).

Previous version

> Both pure tones and harmonic complex tones with 35 frequency components (one band) were employed in control conditions across the experiments.

Revised version

> Both pure tones and (full-band) harmonic complex tones with 35 consecutive harmonics were used as control stimuli (Fig 4) as well as exemplars (Fig 5).

Lines 125–132: Revised as follows (current line 227).

Previous version

> Harmonic complex tones with switching frequency bands were used in experimental conditions. These control and experimental conditions

were used as test sequences to collect data. In addition, to convey the concepts of one stream (integration) and two streams (segregation), four audio exemplars of stimulus sequences were prepared in each experiment: pure-tone sequences and one-band harmonic complex tone sequences, which were separated by 2-semitone intervals (for one stream; H tones with an F or F_0 of 224.5 Hz) and 18-semitone intervals (for two streams; H tones with an F or F_0 of 565.7 Hz).

Revised version

Stripe tones were used as experimental stimuli.

Line 133: The following sentence was added at the beginning of the paragraph (current lines 228–229).

Exemplars' (fundamental) frequency separations were 2 semitones for "one-stream" exemplars and 18 semitones for "two-stream" exemplars.

Line 134: "stimulus type" was replaced with "control stimulus type".

Line 135: "The range of (fundamental) frequency separations was determined referring to Haywood and Roberts [13–15] and the results of our pilot experiment." was added (current lines 233–234).

Lines 135–137: "Thus, H tones had Fs or F_0s of 252, 356.4, and 504 Hz in test sequences. The LHL- tone sequence pattern was used in experiments 1 and 2, and the HLH- pattern was used in experiments 3 and 4." was removed.

Line 138: "experimental" was replaced with "stripe-tone".

Line 139: "frequency bands" was replaced with "bands". Other sections (lines 241, 265, 266, 267, 272, 362, 383, 413, 416, 424, 426, 428, and 434) were revised in the same manner.

Line 139: "(2, 4, 8, and 16 bands)" was replaced with "(2, 4, 8, and 16 bands in Table 1)".

Lines 140–145: "Regarding the number of frequency bands, it refers to the number of passbands in Tables 1 and 2, except for the one band. As a starting point for investigating the effects of switching frequency bands (2–16 bands) on sequential stream segregation with non-speech stimuli, we used the band settings defined by Ueda et al. [66], which were based on the four essential frequency bands in speech perception [67] and critical bands [68]." was removed.

Tables 1 and 2: The "1 band" column was removed.

Table 1, title: "harmonic complex tone stimuli" was replaced with "L-stripe-tones".

Table 2, title: "the" was removed. "in harmonic complex tone stimuli" was replaced with "for stripe tones".

Lines 146–147 "corresponding harmonic numbers in each passband when the F_0 was 200 Hz, i.e., the L-tone F_0" was replaced with "the correspondence between band numbers and harmonic numbers for stripe tones" (current lines 246–247).

Lines 147–155: The following sentences were removed.

These sets of harmonic numbers were maintained for all other stimuli with different F_0s to minimize the variations of pitch salience caused by variation in harmonic number (and resolvability), to fix spectral envelope shapes relative to F_0, and to keep the total number of harmonics constant. Every stimulus in the experimental conditions consisted of either odd-numbered bands or even-numbered bands (both of which contained resolved harmonics, except for those of even-numbered two-band stimuli). These two kinds of stimuli alternated (Figs 2 and 3). Thus, the spectral patterns of the stimuli relative to an F_0 moved upward

when an even-numbered band stimulus followed an odd-numbered band stimulus, and vice versa.

Lines 157–158: "Figs 2(c)–(f) and 3(c)–(f)" was replaced with "Figs 2(a–h) and 3(a–h)." "Figs 2(i)–(l) and 3(i)–(l)" was replaced with "Figs 2(i–p) and 3(i–p)."

Lines 158–163: The following sentences were removed.

Note that "incongruent" does not mean that the spectral patterns of L tones always occupy higher frequency regions than those of H tones in a tone sequence [e.g., Figs 2(l) and 3(l)] because the occupied frequency regions depend on the combinations of F_0s and frequency bands (with corresponding harmonic numbers relative to F_0). Congruent stimuli were used in experiments 1 and 3, and incongruent stimuli were used in experiments 2 and 4.

Lines 163–164: Revised as follows (current lines 265–266).

Previous version

The full sets of stimulus spectrograms are provided in the Supporting information (S1–S6 Figs).

Revised version

For the full set of stimulus spectrograms, see S1–S6 Figs.

Line 164: The following sentences were added (current lines 266–270).

For each experiment, six control conditions [three steps of (fundamental) frequency separations $\times$ two control stimulus types] and 12 stripe-tone conditions (three steps of fundamental frequency separations $\times$ four steps of the number of bands) were constructed. Thus, 18 conditions were prepared for each experiment.

Fig 2: (b) was moved to new Fig 4 as (a). (a), (g) and (h) were removed.

Fig 3: (b) was moved to new Fig 4 as (d). (a), (g) and (h) were removed.

New Fig 4, title: Spectrograms of control stimuli with (full-band) harmonic complex tones.

New Fig 4, caption: LHL_ sequences with (a) 4-, (b) 10-, and (c) 16-semitone separation were used in both experiments 1 and 2. HLH_ sequences with (d) 4-, (e) 10-, and (f) 16-semitone separation were used in both experiments 3 and 4. Pure-tone stimuli were also used but are not shown here.

New Fig 5, title: Spectrograms of audio exemplars with (full-band) harmonic complex tones.

New Fig 5, caption: (a) The LHL_ audio exemplar for one stream (integration) with 2-semitone separation and (b) the LHL_ audio exemplar for two streams (segregation) with 18-semitone separation were used in both experiments 1 and 2. HLH_ exemplars for one stream and two streams (c and d) with 2- and 18-semitone separation were used in both experiments 3 and 4. Note that the 2-semitone separation, as in Fig 5(a and c), was narrower than the 4-semitone separation, which was the narrowest (fundamental) frequency separation used in measuring segregation. Likewise, the 18-semitone separation, as in Fig 5(b and d), was wider than the 16-semitone separation, which was the widest (fundamental) frequency separation used in measuring segregation. Pure-tone audio exemplars were also used (not shown here).

Fig 2, title: Replaced as follows. "Spectrograms of stripe tones with LHL_ triplets."

Fig 2, caption: The whole caption was replaced with the following.

Stripe tones are harmonic complex tones divided into 2, 4, 8, or 16 bands. Frequency components are grouped into several bands based on

harmonic numbers (Tables 1 and 2), and the components in every other band are removed. The tone duration is 80 ms. Two possible spectral patterns (with only odd- or even-numbered bands) are switched between L and H tones to make a sequence. An LHL_ ("_" denotes a "silent gap") triplet is presented three times with 80-ms gaps. The columns are in the order of 2-, 4-, 8-, and 16-band stimuli from left to right. The upper two rows (a–h) represent congruent sequences used in experiment 1 (fundamental frequencies and spectral patterns move in the same direction), and the lower two rows (i–p) represent incongruent sequences used in experiment 2 (fundamental frequencies and spectral patterns move in the opposite direction). The first and third rows from the top (a–d and i–l) represent stimuli with a four-semitone fundamental frequency separation. The second and fourth rows (e–h and m–p) represent stimuli with a 16-semitone separation. The L-tone fundamental frequency is fixed at 200 Hz. The H-tone fundamental frequency for the four-semitone separation is 252 Hz, and the 16-semitone separation is 504 Hz.

Fig 3, title: Replaced as follows. "Spectrograms of stripe tones with HLH_ triplets."

Fig 3, caption: The whole caption was replaced with the following.

> Congruent and incongruent sequences (a–h and i–p) were used in experiments 3 and 4. The columns are in the order of 2-, 4-, 8-, and 16-band stimuli from left to right. The first and third rows (a–d and i–l) represent stimuli with a four-semitone separation, and the second and fourth rows (e–h and m–p) represent stimuli with a 16-semitone separation.

Figs 2 and 3: Redrawn for clarity. S2–S6 Figs were revised similarly.

i. The frequency axis was plotted on a log scale.
ii. The first and the last markers on the time axis were moved to the onset of the first triplet and the offset of the final triplet.
iii. The four values on the time axis were replaced with "0, 320, 640, 880".
iv. "Time (s)" was replaced with "Time (ms)".
v. The numbers or words on each panel were removed, except for those in the leftmost column and the lowest row.
vi. A label of 2 bands, 4 bands, 8 bands, and 16 bands was added for each column from left to right.

Line 166: A paragraph was added at the beginning of the Stimuli section. The sentences in lines 166–167 and 172–174 were moved to the second and first sentences in the new paragraph. Thus, the whole paragraph reads as follows (current lines 272–278).

> All stimuli were generated with custom software written in J language [41], with a sampling frequency of 44100 Hz and a quantization of 16 bits. Harmonic complex tone stimuli were generated by adding equal-amplitude components in sine phase. L (fundamental) frequency was fixed at 200 Hz. H (fundamental) frequencies for 2-, 4-, 10-, 16-, and 18-semitone separations were 225, 252, 356, 504, and 566 Hz. The stimulus and gap duration was 80 ms, including 10-ms rise and fall time with raised-cosine amplitude envelopes for a stimulus.

Line 167: "each tone" was replaced with "each stimulus".

Line 168: The following sentences were added after "calibration tone." (current lines 282–286).

The sound pressure level (SPL) of the calibration tone was adjusted to 70 dB (A). The SPLs at headphone outputs were measured with an artificial ear (Brüel & Kjær type 4153, Brüel & Kjær Sound & Vibration Measurement A/S, Nærum, Denmark), a condenser microphone (Brüel & Kjær type 4192), and a sound level meter (Brüel & Kjær type 2260).

Lines 168–171: Revised as follows (current lines 290–291)

Previous version

Further adjustments to equalize the SPLs of the tones were done by monitoring headphone outputs (we made 60-second tones for monitoring headphone outputs, and applied the adjustments for the tones of 80-ms duration). The level-adjusted tones were ordered and combined with gaps to construct the stimulus sequences.

Revised version

Each stimulus SPL was confirmed with a 60-second tone of the same frequency composition.

(c) The Procedure was streamlined with the following revisions. Moreover, Fig 6 was added to show the schematic illustration of the streaming concept given to participants.

Line 176: ", including instructions, practice trial blocks, and main trial blocks," was removed.

Line 177: "sound-attenuated" was replaced with "sound-attenuating".

Lines 183–186: The descriptions about sound pressure measurement procedures were moved to the Stimuli and revised as follows (current lines 282–286).

The SPLs at headphone outputs were measured with an artificial ear (Brüel & Kjær type 4153, Brüel & Kjær Sound & Vibration Measurement A/S, Nærum, Denmark), a condenser microphone (Brüel & Kjær type 4192), and a sound level meter (Brüel & Kjær type 2260).

Lines 187–193: Revised as follows (current lines 312–314).

Previous version

In the instruction phase, the concepts of one stream and two streams were conveyed through written instructions with schematic illustrations. Also, audio exemplars that demonstrated clear one-stream and two-stream percepts were presented, following the procedure by Haywood and Roberts [13–15], Grimault et al. [20, 26], and Thomassen et al. [7]. The F and F_0 separations of one-stream exemplars (2 semitones) and two-stream exemplars (18 semitones) were outside the range of those in test sequences (4–16 semitones), therefore, participants had to generalize the concept in the experiment.

Revised version

The experimenter explained the concepts of one stream and two streams to the participants using schematic illustrations (Fig 6), written explanations, and audio exemplars.

New Fig 6: A new figure showing the schematic illustrations of the streaming concept was included.

Title: Schematic illustrations given to participants.

Caption: Schematic illustrations for an LHL_ sequence were used in experiments 1 and 2: (a) a test sequence, (b) perception of one stream, and (c) perception of two streams. Similar schematic illustrations were used for an HLH_ sequence in experiments 3 and 4 (d–f). The dashed lines represent

perceptual connections between tones. Participants were instructed to focus on the final triplet (marked with squares) and report whether they heard one stream or two streams. Reproduced with translations from Japanese to English.

Line 196: "(LHL- in experiments 1 and 2; HLH- in experiments 3 and 4) and instructed that we call the percept" was replaced with "called".

Lines 196–198: We elaborated on how the exemplars were presented (current lines 319–323).

Previous version

Then, two one-stream exemplars were presented in the order of pure-tone sequence and one-band harmonic complex tone sequence. A similar procedure was carried out for the two-stream exemplars.

Revised version

Then, pure and full-band tone exemplars with two-semitone separation were presented in this order. Subsequently, they were told that they would hear exemplars sound like a distinctly segregated sequence called "two streams" and presented with pure and full-band tone exemplars with 18-semitone separation.

Line 199: "another" was replaced with "presentations to be repeated".

Lines 199–208: These sentences were moved to just after line 217 as a new paragraph, which now reads as follows (current lines 344–348), along with the following revisions.

Each trial started after a three-second pause to eliminate the build-up effect on stream segregation from previous trials. Each trial block consisted of 18 trials of conditions that were randomly ordered. After two practice trial blocks, 20 main trial blocks were run. Thus, each condition was measured 20 times. Participants were allowed to take a break between the blocks.

Lines 199-201: "On each trial (in the phases of practice trial blocks and main trial blocks), the test sequence was presented once" was replaced with "Each trial started".

Lines 202–205: "The 18 test sequences were randomly ordered in each trial block: six combinations in control conditions (three steps of F_0 separations × two stimulus types) and 12 combinations in experimental conditions (three steps of F_0 separations × four steps of number of frequency bands)." was replaced with "Each trial block consisted of 18 trials of conditions that were randomly ordered."

Line 205: "A total of" was replaced with "After two practice trial blocks,"

Line 205: "were run" was added after "trial blocks".

Lines 205–206: "(each combination" was replaced with "Thus, each condition".

Lines 205–206: ") started after presenting two practice trial blocks" was removed.

Lines 207–208: "Practice trial blocks were prepared so that participants could be familiarized with the task." was removed.

Lines 209–210: "(i.e., the last three tones)" was removed.

Line 210: "three triplet sequences" was replaced with "three-triplet sequences".

Line 211: "two streams" was replaced with "two streams (Fig 6)".

Line 213: ", following the instructions by Haywood and Roberts [13–15]" was removed.

Lines 214–215: Revised as follows (current lines 340–341).

Previous version

to click either the button “One” or “Two” on a computer screen to indicate a response of either one stream or two streams.

Revised version

to select a “One” or “Two” response button on a computer screen.

Lines 216–217: Revised as follows (current line 343).

Previous version

and participants were allowed to cancel and re-enter their responses.

Revised version

providing an opportunity to correct an erroneous input.

Lines 219–220: Revised as follows (current lines 355–356).

Previous version

and took about 60 to 100 minutes for completing each experiment.

Revised version

, taking 60 to 100 minutes to complete each one.

Lines 222–227: The following details were removed.

Three participants were asked to join experiments 1 and 2 again at least 19 days later (before they joined experiments 3 and 4) because we had to fix an issue in the computer program: When a participant double-clicked the response buttons “One” or “Two,” a message box appeared twice. However, sometimes two responses were recorded without presenting the second trial. The previous data for these participants were discarded and replaced with the data obtained with the refined program.

(d) The descriptions in the Statistical method were polished with the following revisions.

Lines 229–230: “To elucidate the effect of independent variables on the binary dependent variable (one stream or two streams) for stream segregation, statistical” was replaced with “Statistical” (current line 367).

Line 232: “experimental” was replaced with “stripe-tone”.

Lines 233–235: “All combinations of independent variables were included in the model to avoid removing the most powerful combinations from the model, even though some combinations might cause extremely high portions of one- or two-stream responses.” was removed.

Line 241: “number of frequency bands” was replaced with “number of bands”.

Lines 246–247: Revised as follows (current lines 394–395).

Previous version

the values of twice the negative of the natural logarithm of the residual pseudo-likelihood function for the model

Revised version

twice the negative of the residual log pseudo-likelihood

Lines 248–249: Revised as follows (current line 396).

Previous version

values of the generalized χ^2 statistic divided by the degrees of freedom

Revised version

ratio of the the generalized χ^2 statistic and its degrees of freedom

(e) The descriptions in the Results were polished with the following revisions.

Line 252: The sentence "Figure 4 shows the results of experiments 1–4." under the section of Control conditions was relocated, immediately following the Results section, and now read as "Figure 7 shows the results of experiments 1–4." (current line 401).

Fig 4 (current Fig 7), caption: The following revisions were made.

i. ", see the corresponding examples of stimulus spectrograms in Figs 2(a)–(f), Figs 2(g)–(l), Figs 3(a)–(f), and Figs 3(g)–(l), respectively" was removed.

ii. "band(s)" was replaced with "bands".

iii. "refer to the frequency band(s)" was replaced with "refer to the number of bands".

iv. "As to pure tones, the abscissa represents F separation." was removed.

v. "nudged" was replaced with "shifted".

Line 264: "(Fig 4)" was removed.

Line 264: "depended on" was replaced with "showed" (current line 429).

Line 265: "By and large" was replaced with "In the congruent conditions [Figs 7(a) and (c)]" (current line 431).

Line 267: ", whereas" was replaced with ". Conversely," (current lines 433–434).

Lines 269–270: ", showing upward-sloping curves in the congruent conditions [Figs 4(a) and (c)]" was removed.

Line 271: "movements" was replaced with "movements [Figs 7(b) and (d)]" (current line 438).

Lines 273 and 277: "eight-" was replaced with "8-".

Lines 274–277: "The segregation responses for the two- and four-band stimuli remained constantly high, irrespective of congruency [Figs 5(a) and (b)], whereas, congruency did affect segregation for the eight- and 16-band stimuli [Figs 5(c) and (d)]." was removed.

Lines 278–279: ", whereas" was replaced with ". In contrast," (current line 447).

Line 281: "(Fig 5)" was removed.

Fig 5 (current Fig 8), caption: The following revisions were made.

i. "To show the effect of congruency on stream segregation with stimuli of 2–16 bands, the percentages of segregation responses in Fig 4" was replaced with "The data in Fig 7".

ii. "with panels" was replaced with "."

iii. "two-, four-, eight-" was replaced with "2-, 4-, 8-".

2.2 Line-to-line Comments

Line 2-4: You may want to make the first two sentences more academic. Also, without mentioning auditory analysis, "frequency" can be confusing.

Response These lines were deleted, and lines 2–19 were revised as the following (current lines 19–43).

Previous version

In our daily lives, we have to group certain frequency (F) components into several objects from a mixture of sounds (integration) and reject others (segregation). Moreover, we have to group a series of sounds into a coherent perceptual stream, which is supposed to come from the same sound source. These general processes of auditory perceptual organization have

been referred to as *auditory scene analysis* [1], including auditory grouping for sequential sounds (e.g., Moore and Gockel [2, 3], Oh et al. [4], Rajasingam et al. [5], Roberts and Haywood [6], and Thomassen et al. [7]) and concurrent sounds (e.g., Gohari et al. [8] and Micheyl and Oxenham [9]). In particular, organizing sequential sounds into two (or more) groups of separate streams has been referred to as *auditory stream segregation* and has been investigated in laboratory experiments with sequentially presented sounds (*sequential stream segregation*).

The majority of the previous investigations on sequential stream segregation have employed pure tones (e.g., Anstis and Saida [10], Bregman and Campbell [11], Bregman [12], Haywood and Roberts [13–15], Miller and Heiser [16], Rajasingam et al. [5, 17], Rankin et al. [18], and van Noorden [19]). Specifically, tone triplet sequences with low-high-low (LHL-) and high-low-high (HLH-) tone triplet patterns have been widely used (Fig 1). Here, L has a frequency (F) lower than that of H, and “-” signifies a silent gap.

Revised version

Auditory scene analysis [1] refers to the auditory function in which we perceptually organize the outer world filled with a mixture of sounds. Sometimes, we can exploit binaural cues to separate several sounds, but the cues are not always available or practical. Even in such situations, normal-hearing listeners usually successfully pick up a target from the background sounds, like a talker’s voice talking on a noisy railway platform over a phone. Linguistic cues should contribute a lot to segregating speech and background noise in such a situation; nevertheless, the perceptual characteristics of the auditory system should be involved in the segregation process. Furthermore, if both a target and background are nonspeech, like a warning signal in background noise, we must make use of other cues, such as pitch and timbre. The auditory system can organize and segregate a sound mixture into more than two streams with the pitch and timbre differences of the sounds. The current investigation focuses on how pitch and timbre interact in *auditory stream segregation* [2].

Miller and Heise [3] reported that two rapidly alternating pure tones (i.e., tones having only one frequency component and a sinusoidal waveform) with some slight frequency differences are heard as a trill, a continuously changing pitch pattern. However, the two tones with a wide frequency separation are perceived as two separate and unrelated melodies. Since then, *sequential stream segregation* has been a widely studied research topic [4–22]. In particular, low-high-low (LHL_) and high-low-high (HLH_) tone patterns, i.e., so-called *triplets* (Fig 1), are the standard research tools to investigate sequential stream segregation caused by pitch differences. Here, “L” signifies a “low-pitch tone,” “H” a “high-pitch tone,” and “_” a “silent gap”. In this connection, we define a “(fundamental) frequency separation” as a “(fundamental) frequency difference between a low-pitch tone and a high-pitch tone, expressed in semitone units” in this article.

Line 21: “Whereas, if Ls and Hs are perceptually grouped into one stream,” do you mean “L-H-L-H-...”?

Response Yes, we do. The confusion was caused by using “-” (a hyphen) to represent

both the connecting symbol in the orthography and a silent gap. We replaced the sign representing a silent gap with "_" (an underscore) thereafter to avoid confusion. Also, the descriptions in lines 16–23 were improved as follows (current lines 37–43 and 48–52).

Previous version

> Specifically, tone triplet sequences with low-high-low (LHL-) and high-low-high (HLH-) tone triplet patterns have been widely used (Fig 1). Here, L has a frequency (F) lower than that of H, and "-" signifies a silent gap. For an example with the LHL- tone triplet sequence, when Ls are perceptually grouped with a short gap in between (L-L-L-L-L-L...) and Hs with a long gap in between (-H—H—H–...), these two streams are segregated. Whereas, if Ls and Hs are perceptually grouped into one stream, a galloping rhythm pattern (LHL-LHL-LHL-...) is perceived.

Revised version

> In particular, low-high-low (LHL_) and high-low-high (HLH_) tone patterns, i.e., so-called *triplets* (Fig 1), are the standard research tools to investigate sequential stream segregation caused by pitch differences. Here, "L" signifies a "low-pitch tone," "H" a "high-pitch tone," and "_" a "silent gap". In this connection, we define a "(fundamental) frequency separation" as a "(fundamental) frequency difference between a low-pitch tone and a high-pitch tone, expressed in semitone units" in this article.
>
> Suppose a series of "LHL_" triplets is presented. If the tone sequence is perceptually grouped, then a listener will hear one stream with a galloping rhythm (LHL_LHL_LHL_). In contrast, if the tone sequence is perceptually segregated into two streams, a listener will hear a low-pitch-tone sequence with a short gap in between (L_L_L_...) and a high-pitch-tone sequence with a long gap in between (H___H___H____...).

Line 23: The introduction can be improved by stating the theoretical and practical importance of segregation–why do we need to promote it?

Response "To promote segregation" is not the purpose of this investigation, but the conditions that promote segregation have theoretical importance. We realized that we must provide an appropriate context to understand this point, therefore, we rewrote the first paragraph of the Introduction (lines 2–12). Furthermore, the sentences explaining the conditions that promote segregation (lines 23–34) were totally rewritten as a new paragraph as the following (current lines 53–65).

Previous version

> One way to promote segregation is to widen the F separation between Ls and Hs [19], and the other way is to prolong a sequence (*building-up* [10, 12]). With an induction sequence prior to a test sequence consisting of three sets of LHL- tone triplets, Haywood and Roberts [13–15] showed the build-up in sequential stream segregation by examining participants' responses to the final triplets (i.e., the last three tones). At the same time, only three sets of triplets (their control conditions) may be segregated with prominent F separation [13–15, 18]. Haywood and Roberts [13–15] revealed that a separation of 14 semitones between L and H tones caused around 75–90% segregation, while a separation of four semitones caused almost no segregation. With a

10-semitone separation, the percentages of segregation responses came in between (around 25–55%). Thus, the effect of F separation on sequential stream segregation was shown with this convenient paradigm of three sets of triplets.

Revised version

One possibility to increasing probability of segregation is to widen the frequency separation between Ls and Hs [4], and the other possibility is to prolong a sequence to *build up* segregation [5, 6]. With an induction sequence prior to a test sequence consisting of three sets of LHL_ tone triplets, Haywood and Roberts [13–15] reported a build-up in sequential stream segregation in participants' responses to the final triplets. At the same time, with only three sets of triplets, they found that a separation of 14 semitones between L and H tones caused segregation around 75%–90% of the time, while a separation of four semitones caused almost no segregation. With a 10-semitone separation, the percentages of segregation responses came in between (around 25%–55%).

Line 35: You may want to explain pure vs. harmonic complex tones to the naive audience.

Response Pure tones were introduced as "(i.e., tones having only one frequency component and a sinusoidal waveform)" at the beginning of the second paragraph in the Introduction. The first two sentences of the third paragraph (lines 35–37) in the Introduction were revised to introduce harmonic complex tones as follows (current lines 67–77).

Previous version

Some studies on sequential stream segregation have used harmonic complex tones. Both fundamental frequency (F_0) separation and spectral differences, which roughly correspond to pitch and timbre differences, affect stream segregation.

Revised version

Harmonic complex tones have been used in some studies on sequential stream segregation. A harmonic complex tone consists of a series of pure tones called harmonics with frequencies that are integer multiples of a fundamental frequency, i.e., the lowest frequency. The pitch of a harmonic complex tone usually corresponds to the pitch of the fundamental; however, even if the fundamental frequency component is missing, the pitch of the harmonic complex tone does not change. This is called the missing fundamental phenomenon. When one modifies the level balance of frequency components in a harmonic complex tone, timbre varies, although this is not the only factor that affects the timbre of a harmonic complex tone.

Line 39: In fact, it is from this line that I realised the importance of F segregation. You may want to move this up. (See my comment on Line 23.)

Response Assuming you meant "frequency separation" by "F segregation," we believe the revisions made for the third paragraph in the Introduction (lines 35–37) improved readers' understanding.

Line 95: I am not sure about the statistical power of this sample size, especially since their music-training and listening-training backgrounds are complex. Also, any gender difference?

Response We have addressed these issues as follows.

1. Statistical power was reported (current lines 416–427 and 459–480).
2. “Extracurricular musical training” was included as candidate random effects in our GLMM analysis with the revisions in lines 240 and 243–245. However, there is no reason to believe that gender affects current results. Moreover, introducing this effect as a random effect did not improve model fitting. Therefore, we decided not to describe the effect of gender in the manuscript.
3. The detailed descriptions of Technical Listening Training classes were removed because it is impossible to discuss the effects of training without a control group in this respect. Also, the general training effect of the Technical Listening Training classes has never been examined in formal experiments with control groups.

Line 101: Absolute pitch, in my understanding, is quite important to the results of the present studies. A short test rather than self-reporting data will improve the validity of the results.

Response You are correct; however, we could not conduct an additional absolute pitch test because most of these participants were unavailable anymore.

Line 128: How were the exemplars introduced to the audience?

Response We elaborated it with the following revisions.

Lines 187–193: Revised as follows (current lines 312–314).

Previous version

In the instruction phase, the concepts of one stream and two streams were conveyed through written instructions with schematic illustrations. Also, audio exemplars that demonstrated clear one-stream and two-stream percepts were presented, following the procedure by Haywood and Roberts [13–15], Grimault et al. [20, 26], and Thomassen et al. [7]. The F and F_0 separations of one-stream exemplars (2 semitones) and two-stream exemplars (18 semitones) were outside the range of those in test sequences (4–16 semitones), therefore, participants had to generalize the concept in the experiment.

Revised version

The experimenter explained the concepts of one stream and two streams to the participants using schematic illustrations (Fig 6), written explanations, and audio exemplars.

New Fig 6: A new figure showing the schematic illustrations of the streaming concept was included.

Title: Schematic illustrations given to participants.

Caption: Schematic illustrations for an LHL_ sequence were used in experiments 1 and 2: (a) a test sequence, (b) perception of one stream, and (c) perception of two streams. Similar schematic illustrations were used for an HLH_ sequence in experiments 3 and 4 (d–f). The dashed lines represent perceptual connections between tones. Participants were instructed to focus on the final triplet (marked with squares) and report whether they heard one stream or two streams. Reproduced with translations from Japanese to English.

Line 196: “(LHL- in experiments 1 and 2; HLH- in experiments 3 and 4) and instructed that we call the percept” was replaced with “called”.

Lines 196–198: We elaborated on how the exemplars were presented (current lines 319–323).

Previous version

Then, two one-stream exemplars were presented in the order of pure-tone sequence and one-band harmonic complex tone sequence. A similar procedure was carried out for the two-stream exemplars.

Revised version

Then, pure and full-band tone exemplars with two-semitone separation were presented in this order. Subsequently, they were told that they would hear exemplars sound like a distinctly segregated sequence called "two streams" and presented with pure and full-band tone exemplars with 18-semitone separation.

Line 199: "another" was replaced with "presentations to be repeated".

Line 326: "Participants might have misunderstood..." Is this misunderstanding purposefully? Is it that they could not help but base their responses on pitch perception? If it is the latter, you may want to discuss the auditory/cognitive indication of this mistake.

Response No, this misunderstanding is not purposeful. We removed the section because the arguments are trifles anyway.

Line 375: "Thus, the combinations of these three variables affected sequential stream segregation." This sentence is important but could be illustrated more. What do you mean by "combinations," and what are the real-world implications?

Response Thank you very much for pointing this out. We realized that the "combinations" are the same as "interactions" therefore the whole paragraph essentially repeats the results. We removed the sentences in lines 363–375, connected the first sentence of the paragraph to the next paragraph, and replaced "In other words," with "This means that" at the beginning of the next paragraph (current line 575).

Line 396-397: "A transition from a segregated percept to an integrated percept was realised by increasing the number of frequency bands for the stimuli" sounds like an improvement. Is it an improvement? If not, please be careful with the tone of description in the manuscript.

Response No, it is not an improvement. The sentence was revised as "Manipulating the number of bands was reflected in the percentages of segregation responses." (current lines 596–597).

3 Corrections and Modifications Made by the Authors

Line 102: "5.8 years" was corrected with "10.1 years" (current line 195). The previous calculation was wrong because the first author excluded data from the years of compulsory musical education, regardless of whether participants had extracurricular musical training during that time.

Table 1, caption: The reference "Ueda *et al.* (2022)" was replaced with "Ueda *et al.* (2023)".

Line 408: "Strictly speaking, it" was replaced with "It".

Line 409: "whether or not" was replaced with "whether".

Line 416: "(i.e., spectral envelope shapes getting closer)" was removed.

Lines 418–419: "depending on the F_0 separations." was replaced with "depending on the fundamental frequency separations and thus pitch separations." (current lines 657–658).

Line 424: “two to 16 frequency bands” was replaced with “2 to 16 bands based on harmonic numbers” (current lines 664–665).

Line 431: “does capture spectral patterns rapidly changing at every 80 ms” was replaced with “captures rapidly changing spectral patterns” (current lines 671–672).

Line 433: “harmonics distinctly” was replaced with “frequency components”.

Lines 477–478: “the J language software routine” was replaced with “J language software routines” (current lines 680–681).

Reference no. 19 (current reference no. 4): “[Ph.D. Dissertation].” was replaced with “. Ph.D. Thesis,”.

Reference no. 37 (current reference no. 35): The name of the third author “Rich Zendel B.” was replaced with “Zendel BR.”.

Reference no. 70 (current reference no. 42): “Version 9.0 [computer language]; 2018.” was replaced with “Version 9.6.3 [computer language]; 2021.”

Reference no. 71 (current reference no. 43): We performed statistical analyses with a new version of the software during the revision process of this paper; therefore, “Version 17.2.0 [computer program]; 2023” was replaced with “Version 18.1.0 [computer program]; 2024”.

References no. 69–71 (current references no. 41–43): The period symbol at the end of the URL was removed.

Supporting information: The whole section was moved to the end of the manuscript according to the submission guidelines.

S1 and S2 Figs: S1 Fig was replaced with a new figure. (a–c) and (d–f) in S1 Fig were moved to S2 Fig as (a–c) and (g–i). (a–c) and (d–f) in S2 Fig were moved to (d–f) and (j–l).

New S1 Fig, title: Spectrograms of audio exemplars.

New S1 Fig, caption: LHL_ triplets with pure tones and full-band harmonic complex tones (a–b and c–d) were used in both experiments 1 and 2. HLH_ triplets with pure tones and full-band harmonic complex tones (e–f and g–h) were used in both experiments 3 and 4. The left column represents exemplars with 2-semitone separation for explaining the one-stream concept, and the right column represents exemplars with 18-semitone separation for explaining the two-stream concept.

S2 Fig, title: “the one-band harmonic complex tone stimuli” was replaced with “control stimuli”.

S2 Fig, caption: Revised as “LHL_ triplets with pure tones and full-band harmonic complex tones (a–c and d–f) were used in both experiments 1 and 2. HLH_ triplets with pure tones and full-band harmonic complex tones (g–i and j–l) were used in both experiments 3 and 4. The columns are in the order of 4-, 10-, and 16-semitone separation from left to right.”

S2–S6 Figs: Legends of “4 semitones”, “10 semitones”, and “16 semitones” were added for the left, center, and right columns, in this order.

S3 Fig, title: “the two-band stimuli” was replaced with “two-band stripe tones”.

S4 Fig, title: “the four-band stimuli” was replaced with “four-band stripe tones”.

S5 Fig, title: “the eight-band stimuli” was replaced with “eight-band stripe tones”.

S6 Fig, title: “the 16-band stimuli” was replaced with “16-band stripe tones”.

S3–S6 Figs, caption: Revised as “LHL$_{-}$ triplets with congruent and incongruent stripe tones (a–c and d–f) were used in experiments 1 and 2. HLH$_{-}$ triplets with congruent and incongruent stripe tones (g–i and j–l) were used in experiments 3 and 4.”

S1 and S2 Tables, title: “GLMM summary” was replaced with “GLMM analysis summary”.

References

Shearer, D. E., Molis, M. R., Bennett, K. O., and Leek, M. R. (**2018**). “Auditory stream segregation of iterated rippled noises by normal-hearing and hearing-impaired listeners,” J. Acoust. Soc. Am. **143**(1), 378–387.

Ueda, K., Doan, L. L. D., and Takeichi, H. (**2023**). “Checkerboard and interrupted speech: Intelligibility contrasts related to factor-analysis-based frequency bands,” J. Acoust. Soc. Am. **154**(4), 2010–2020.

Ueda, K., Takeichi, H., and Wakamiya, K. (**2022**). “Auditory grouping is necessary to understand interrupted mosaic speech stimuli,” J. Acoust. Soc. Am. **152**(2), 970–980.
